# Structural basis for the recognition of diastereomeric 5′,8-cyclo-2′-deoxypurine lesions by the human nucleotide excision repair system

**DOI:** 10.1093/nar/gku162

**Published:** 2014-03-10

**Authors:** Konstantin Kropachev, Shuang Ding, Michael A. Terzidis, Annalisa Masi, Zhi Liu, Yuqin Cai, Marina Kolbanovskiy, Chryssostomos Chatgilialoglu, Suse Broyde, Nicholas E. Geacintov, Vladimir Shafirovich

**Affiliations:** ^1^Department of Chemistry New York University, 100 Washington Square East, New York, NY 10003, USA, ^2^Department of Biology, New York University, 100 Washington Square East, New York, NY 10003, USA and ^3^Istituto per la Sintesi Organica e la Fotoreattività, Consiglio Nazionale delle Ricerche, Via P. Gobetti 101, 40129 Bologna, Italy

## Abstract

The hydroxyl radical is a powerful oxidant that generates DNA lesions including the stereoisomeric *R* and *S* 5′,8-cyclo-2′-deoxyadenosine (cdA) and 5′,8-cyclo-2′-deoxyguanosine (cdG) pairs that have been detected in cellular DNA. Unlike some other oxidatively generated DNA lesions, cdG and cdA are repaired by the human nucleotide excision repair (NER) apparatus. The relative NER efficiencies of all four cyclopurines were measured and compared in identical human HeLa cell extracts for the first time under identical conditions, using identical sequence contexts. The cdA and cdG lesions were excised with similar efficiencies, but the efficiencies for both 5′*R* cyclopurines were greater by a factor of ∼2 than for the 5′*S* lesions. Molecular modeling and dynamics simulations have revealed structural and energetic origins of this difference in NER-incision efficiencies. These lesions cause greater DNA backbone distortions and dynamics relative to unmodified DNA in 5′*R* than in 5′*S* stereoisomers, producing greater impairment in van der Waals stacking interaction energies in the 5′*R* cases. The locally impaired stacking interaction energies correlate with relative NER incision efficiencies, and explain these results on a structural basis in terms of differences in dynamic perturbations of the DNA backbone imposed by the *R* and *S* covalent 5′,8 bonds.

## INTRODUCTION

**Figure 1.**
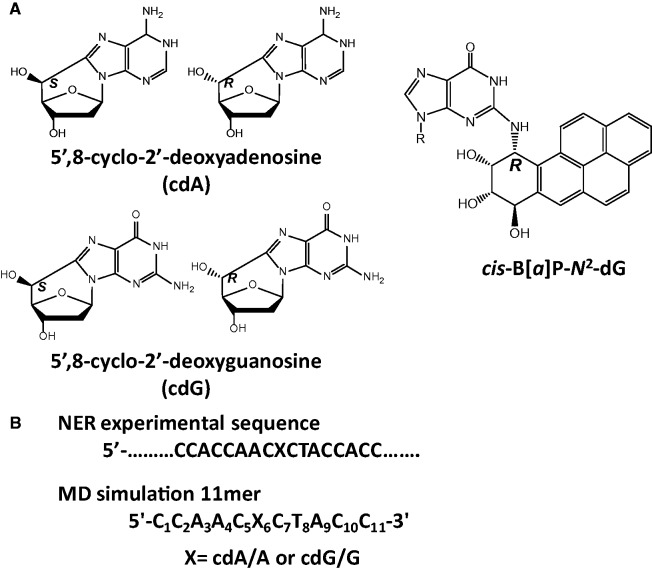
(**A**) Chemical structures of the cdA and cdG stereoisomeric lesions, together with the structure of the 10*R* (+)-*cis*-B[*a*]P-*N*^2^-dG NER activity standard used to compare activities for cell extracts prepared at different times. (**B**) Sequences of the B-DNA duplexes used in the experimental and MD simulation studies. X denotes the modified site. For control simulations with unmodified duplexes, X = A or G. The complementary partner strands contain T opposite cdA/A and C opposite cdG/G.

The generation of hydroxyl radicals (1,2) and other reactive species generated during the inflammatory response gives rise to the formation of a variety of genotoxic DNA lesions (3). The reactive intermediates include free oxyl radicals such as 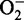, 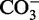 and OH (4). The 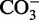, NO_2_ and OH are powerful oxidizing agents, but only hydroxyl radicals (5) and carbonate radical anions (6) can directly abstract electrons from nucleic acid bases in DNA. The superoxide radical itself is not an oxidant, but in protic environments its dismutation gives rise to O_2_ and H_2_O_2_, with the latter being a potential source of OH radicals (7). The reactions of OH radicals with DNA can occur by two pathways that include either attack on nucleobases, or hydrogen atom abstraction from 2-deoxyribose residues (8,9). The selectivity of H-atom abstraction is correlated with the solvent-exposure of the 2-deoxyribose H-atoms, and the abstraction of H5′ atoms is dominant (55%) (10). In contrast to other carbon-centered 2-deoxyribose radicals, the C5′ radicals do not generate abasic sites but form unique 5′,8-cyclopurine-2′-deoxyadenosine (cdA) and -2′-deoxyguanosine (cdG) lesions [cdA and cdG, respectively (11,12)]. The formation of these lesions occurs by the addition of the C5′-radical to the C8-positions of A or G bases, followed by the one-electron oxidation of the resulting N7-radicals by weak oxidants such as molecular oxygen, to produce the cdA and cdG end products (13–15). Both cdA and cdG lesions exist as 5′*R*- and 5′*S*-diastereomers (Figure 1) and are formed in proportions that depend on the solvent and DNA conformation (16–23).

The cdA and cdG lesions have been found in γ-irradiated human-cultured cells (17,24), in humans (22), and in tissues of Long-Evans Cinnamon rats (25). The cyclopurines are highly mutagenic (26,27) and their accumulation in cellular DNA may contribute to the etiology of cancer and other diseases (24,28–33). In contrast to most oxidatively generated DNA lesions, cyclopurines are not substrates of base excision repair (BER), but are good substrates of the mammalian nucleotide excision repair (NER) system (34–36). However, the efficiencies of dual incisions are considerably lower than in the case of *cis*-Pt G*TG* intrastrand cross-links (35). The prokaryotic NER apparatus also removes cyclopurine lesions, albeit inefficiently (37). Kuraoka *et al.* (35) reported that the NER dual incision efficiencies are about four times greater for the 5′*R*- than the 5′*S*–cdA lesion incorporated into plasmid DNA in human HeLa cell extracts. The NER incision efficiencies for removing 5′*S*–cdG lesions in HeLa cell extracts were found to be ∼1.5 times greater than the repair of the 5′*S*–cdA lesion in 135-mer duplexes (36). However, the relative incision efficiencies of all four of these 5′,8-cyclopurine-2′-deoxynucleotide lesions have never been compared under identical experimental conditions, in identical sequence contexts and in the same mammalian cell extracts; these extracts usually exhibit variable NER activities, thus making it difficult to compare NER activities between different experiments and in different laboratories. Furthermore, the susceptibility to mammalian NER of the 5′*R*–cdG lesion has never been presented.

Both the mammalian and the prokaryotic NER systems are widely believed to recognize the distortions/destabilizations caused by DNA lesions rather than the chemical nature of the lesions (38–41). Many bulky lesions cause significant destabilizing distortions to the DNA and therefore are good substrates of NER (42,43). Examples for mammalian NER are DNA adducts derived from bulky hetero-aromatic amines such as the acetylated and unacetylated 2-aminofluorene derivatives (44,45), other polycyclic aromatic diol epoxide metabolites (42,46,47), UV photoproducts (48) and cisplatin adducts (49). However, the origins of the recognition and susceptibility of the relatively small 5′,8-cyclopurine-2′-deoxynucleotide lesions to the mammalian NER system are poorly understood. The presently available NER data is based on dual incision experiments performed with 5′*S*–cdA, 5′*S*–cdG and 5′*R*–cdA lesions in three different laboratories in different sequence contexts (34–36), and no data on the NER susceptibility of the 5′*R–*cdG lesion has been reported.

The objectives of this work were to examine the structural features of the 5′,8-cyclopurine lesions that govern their susceptibility to NER and that lead to differences in incision of the 5′*R* and 5′*S* stereoisomers. For this purpose, it was necessary to compare the relative NER efficiencies of the pairs of 5′*R* and 5′*S* cdA and cdG lesions under the same conditions, in identical sequence contexts, and in the same cell extracts. The structural and energetic factors that characterize these four different lesions in double-stranded DNA were examined using modeling and molecular dynamics simulation (MD) methods that are based on published NMR-solution structures of the 5′*S*–cdG (50) and 5′*S*–cdA (51) lesions. These simulations provide detailed insights into the origins and the stereochemical dependence of the NER susceptibilities of the four different 5′,8-cyclonucleotide lesions in DNA.

## MATERIALS AND METHODS

### Experimental methods

#### Synthesis of oligonucleotides containing single cdG or cdA lesions

Phosphoramidites of the four different nucleosides of *R* and *S* cdA and cdG (Figure 1) were prepared following the radical-based protocols developed previously (20,52). The site-specifically modified 17-mer oligo-2′-deoxyribonucleotide (ODN) sequence 5′-d(CCACCAAC[X]CTACCACC) with X = 5′*R*– or 5′*S*–cdG/cdA were synthesized following previously published procedures (53,54). After standard deprotection with AMA reagent [NH_4_OH (30%)/CH_3_NH_2_ (40%)] the crude 5′-DMTr-on ODNs were detritylated and purified by reversed phase HPLC, as described (54). Further purification was carried out by strong anion-exchange (SAX) HPLC and then by PAGE. The purity and homogeneity of the collected fractions were controlled by SAX-HPLC and analytical gel electrophoresis. The molecular weights of the ODNs were assessed by MALDI-TOF in the negative mode.

### Thermal stabilities of modified and unmodified oligonucleotide duplexes with single cdG or cdA lesions

The 5′,8-cyclonucleotide-containing and unmodified homologous 17-mer ODN strands (5′-d(CCACCAAC[X]CTACCACC) with X = cdA or cdG) were mixed in equimolar amounts with the complementary strands in 0.1 M NaCl, 20 mM sodium phosphate, pH 7.0 buffer solution. The oligonucleotide strands were annealed by heating equimolar solutions to 85°C and cooling overnight. The melting curves were determined by monitoring the absorbance of the solutions at 260 nm as a function of temperature measured by a Cary 100 spectrophotometer system equipped with a temperature controller using a 0.4°C/min heating rate. The melting points, *T_m_*, were determined from the half-maximum between the low- and high-temperature limits of the melting curves.

### Preparation of cdG/cdA-containing 147-mer DNA duplexes

The NER assays were conducted with 147-mer DNA duplexes (Supplementary Figure S1) that were prepared by ligation methods using T4 ligase (USB Molecular Biology Reagents and Biochemicals, Cleveland, OH) as described elsewhere (55). Briefly, the 17-mer long oligonucleotides 5′-d(CCACCAAC[X]CTACCACC) were 5′-endlabeled with [γ-^32^P]ATP (6000 Ci/mmol, PerkinElmer Life Sciences, Boston, MA) and incorporated into a 147-mer duplex as described in Supporting Information. Two different 147-mer ODNs were available from other experiments being conducted in the laboratory and were adopted for the present studies. These two ODNs, termed ODN(1) and ODN(2) were used in order to enhance the statistical accuracies of the relative dual incision efficiencies of different lesions studied in this work. In both ODNs, X was embedded in the same 17-mer sequence context, but at either the 66th ODN(1) or the 70th nucleotide position ODN(2) from the 5′-end. The full sequences are described and additional information is provided in Supplementary Material. These internally and radioactively labeled 147-mers were purified using 12% denaturing polyacrylamide gels and were subsequently annealed with their fully complementary 147-mer strands by heating the solutions to ∼90°C for 2 min and cooling overnight to 4°C.

As a relative measure of the NER activities of different cell extracts, each set of experiments included a 147-mer oligonucleotide containing the stereochemically defined 10*R* (+)-*cis-anti*-B[*a*]P-*N*^2^-dG adduct (abbreviated as *cis*-B[*a*]P-*N*^2^-dG) that was used as a positive control and reference standard of NER activity with different cell extracts prepared at different times (56).

### NER assays experiments

The cell extracts were prepared using standard methods (57) that were slightly modified as described (58). In each set of experiments, the different 147-mer duplexes that included unmodified controls, and duplexes with either the *cis*-B[*a*]P-*N*^2^-dG or one of the four 5′,8-cyclopurine lesions, were incubated in 80 µl aliquots of the same cell extracts (containing 60–80 µg of protein) for varying amounts of time. Following incubation, the oligonucleotide excision products and intact DNA were desalted by precipitation with an aqueous 80% methanol solution and subjected to denaturing 12% polyacrylamide gel electrophoresis. The dried gels were then analyzed by gel autoradiography using a Storm 840 phosphorimager.

### Modeling and MD simulations

We built molecular models of the 5′*S* and 5′*R* cdA and cdG lesions in the sequence shown in Figure 1B based on the coordinates of the NMR-solution structures for the 5′*S–*cdA (PDB ID: 2LSF (51,59) and 5′*S–*cdG lesions (PDB ID: 2LFA) (50,59). We excised the 5′*S–*cdA and 5′*S*–cdG nucleosides, and optimized their geometries with Gaussian 03 (60). We built a B-DNA model for the two sequences shown in Figure 1 and replaced the adenine or guanine with the 5′*S–*cdA and 5′*S–*cdG. To create the 5′*R* stereoisomers, we inverted the chirality at the C5′ of the 5′*S* nucleosides and then optimized the 5′*R* nucleosides with Gaussian 03, and replaced the unmodified adenine or guanine with the 5′*R* lesions. InsightII (Accelrys Software, Inc.) was used for the modeling. Subsequently, we carried out 100 nanoseconds (ns) of production MD simulations for the 5′*R*– and 5′*S*–cdA and cdG lesions and their respective unmodified controls (Figure 1). We used AMBER12 (61) with the Cornell *et al.* force field (62) and the Parm99.dat parameter set (63) modified by parmbsc0 (64). The force fields for the cyclopurine lesions were custom parameterized to be consistent with the AMBER force field and parameters are given in Supplementary Tables S1. The bond, angle and dihedral parameters are the same for the 5′*R* and 5′*S* stereoisomers. Equilibrium bond angles *θ*_eq_ (Supplementary Table S1) were assigned from the QM optimized structures. However, separate partial charges had to be developed for the 5′*S* and 5′*R* cdA and cdG nucleotides (Supplementary Tables S2 and S3). The partial charges for the modified nucleotides were computed utilizing quantum mechanical Hartree Fock calculations with the 6-31G* basis set using Gaussian 03. The charges were then fitted to each atomic center with the RESP algorithm (62,65). The details of the MD protocol are given in Supplementary Material. RMSD values of the current structure relative to the initial structure of the production MDs are shown in Supplementary Figure S2. The last 70 ns of the production MD were utilized for all the analyses. We used the ANAL module of AMBER to calculate van der Waals interaction energies between the cdA:T or cdG:C base pair or the respective unmodified A:T or G:C pairs and their adjacent base pairs in the central trimers of Figure 1B; the energy differences between the lesioned and the respective unmodified cases were then computed. Helical parameters were computed with Toolchest (66,67). Standard deviations of block averages were computed using the block averaging method (68), and the block size was determined using the convergence of the standard deviation of the block averages (69). Details are given in Supplementary Material. The best representative structures were obtained using the clustering command in the Ptraj module of AMBER 11 using the RMSD similarity metric. PyMOL (70) was employed to make molecular images and movies. For Movie S5, we selected five frames from the MD simulations that represent extreme and intermediate states in the dynamics of the 5′*R–* and 5′*S*–cdA lesions.

## RESULTS AND DISCUSSION

### NER efficiencies of 5′,8-cyclopurine-2′-deoxynucleotide lesions in double-stranded DNA duplexes: 5′*R* stereoisomers are better excised than 5′*S*

Pairs of 147-mer oligonucleotide duplexes ODN(1) and ODN(2) containing one of the four 5′,8-cyclopurine-2′-deoxynucleotide lesions were incubated with aliquots of the same cell extracts. In each case, a 147-mer duplex containing a single *cis*-B[*a*]P-*N*^2^-dG adduct was also incubated with another portion of the same cell extract; the fractions of duplexes that successfully underwent the dual incision reaction served as a control for the variable NER activities of cell extracts prepared on different days (56). All results obtained with the 5′,8-cyclopurine-2′-deoxynucleotide lesions embedded in 147-mer duplexes were normalized to the fractions of dual incisions observed for the *cis*-B[*a*]P-*N*^2^-dG sample at the 30-min incubation time point.

**Figure 2.**
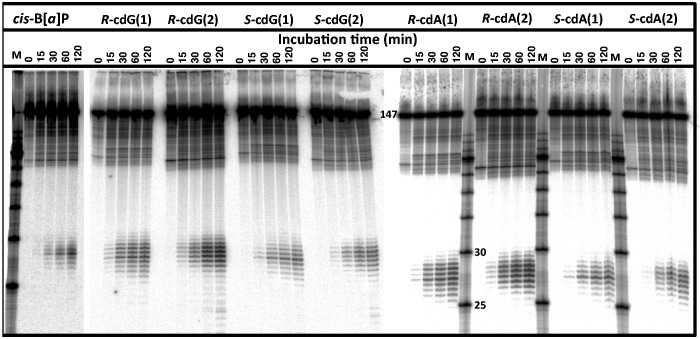
NER in HeLa cell extracts. Typical denaturing gels are shown that illustrate the appearance of dual incision products elicited by the *cis*-B[*a*]P-*N^2^*-dG adduct (positive control) and the 5′*R*–cdG, 5′*S*–cdG, 5′*R*–cdA and 5′*S*–cdA lesion-containing 147-mer duplexes as a function of incubation time. The lanes M represent oligonucleotide size markers: the 25 and 30-mer bands are marked. All of the results shown were conducted with the same HeLa cell extract, but three separate electrophoresis gels (*cis*-B[*a*]P, cdG and cdA) were used in order to visualize the dual incision products. Each set of experiments was conducted with the ODN(1) and ODN(2) duplexes that are almost identical since the same cyclopurine lesions were embedded in nearby different positions in the 147-mer duplexes (see the text and Supplementary Material for details). These two oligonucleotides were employed for enhancing the statistical significance of the results.

**Figure 3.**
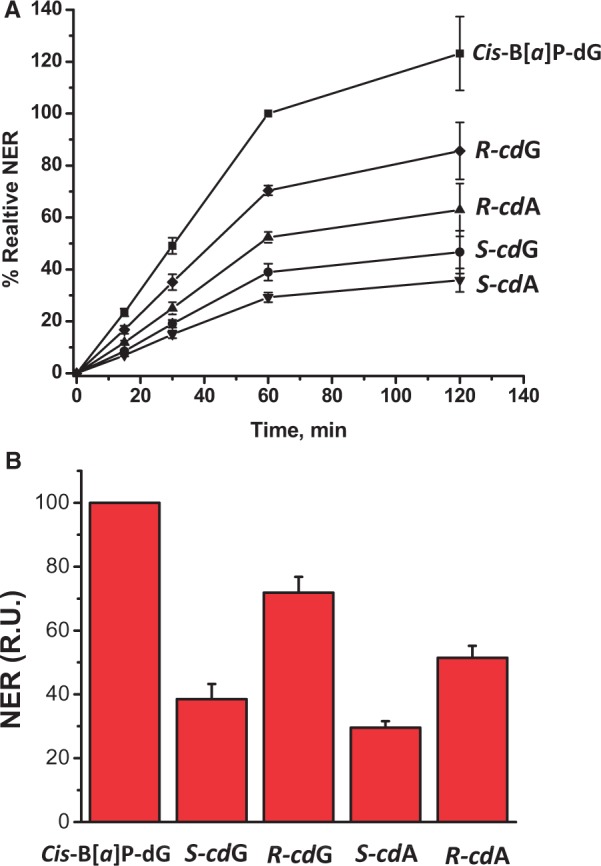
(**A**) Time course of NER dual incision product formation of 24–32 oligonucleotide fragments bearing either the *cis*-B[*a*]P-dG, 5′*R*–cdG, 5′*R*–cdA, 5′*S*–cdG or 5′*S*–cdA lesions. The experimental data points are averages of five independent experiments in different cell extracts, and the error bars represent the standard deviations. The incision efficiencies of the cdG- and cdA-containing sequences were normalized in each of the five independent experiments to the value obtained with *cis*-B[*a*]P-dG (relative value of 100 at the 60-min time point, also determined in each experiment). The results are thus provided in relative units (RU). The reaction kinetics are linear up to at least 60 min incubation times, and rates of reaction level off within the 60–120-min time interval. (**B**) Relative initial rates of dual incision kinetics determined by averaging the slopes of the straight line portions of the data obtained with the ODN(1) duplex (panel A), and the analogous plot obtained with the ODN(2) duplex (Supplementary Figure S3). The results obtained with the ODN(1) and ODN(2) duplexes were the same within experimental error (Supplementary Figure S3), and the 17-mer sequence contexts in which the cyclopurine lesions are embedded, are identical in ODN(1) and ODN(2).

A typical set of results showing the appearance of dual incision products of all four 5′,8-cyclopurine-2′-deoxynucleotides embedded in DNA duplexes ODN(1) or ODN(2) as a function of incubation time are shown in Figure 2. The yields of dual incisions observed in the same cell extract with the *cis*-B[*a*]P-*N*^2^-dG reference adduct are also shown; the 60-min incubation time point was used as the reference NER efficiency (arbitrary value of 100). Densitometric analysis of the phosphorimager results were used to evaluate the radioactivities in the 24–32-mer oligonucleotide excision product range, and the total radioactivity in a given lane (Figure 2). In all cases, the fractional yields of dual incision products increased linearly as a function of incubation time up to 60 min (Figure 3A), but the rates became markedly slower within the 60–120-min time interval. Analogous results were obtained with the ODN(2) sequence (Supplementary Figure S3). Thus, the 120-min time points shown in Figures 2 and 3A were not considered in the analysis of incision rates. The slopes of the linear plots in the 0–60-min time interval reflect the relative initial rates of NER dual incisions of the different substrates (Figure 3B). The 5′*R*-stereoisomers of either cdA or cdG are recognized ∼2-fold better than the 5′*S*-stereoisomers. While the cdG lesions appear to be somewhat better substrates than the cdA lesions for each stereoisomer, the differences are more modest and closer to experimental error, particularly in the case of the *S*-stereoisomers. Thus, the stereochemical properties of the lesions, rather than the nature of the purines, primarily influence the NER dual incision rates in our sequence context.

These measured relative NER efficiencies are qualitatively consistent with the observations of Kuraoka *et al.* (35) who reported a higher (5′*R*–cdA)/(5′*S–*cdA) NER efficiency ratio (factor of ∼4) and Pande *et al.* (36) who obtained 5′*S*–cdG/5′*S*–cdA excision ratios of ∼1.3–1.5. The quantitative differences between these published values and those reported here are most likely attributable to base sequence context effects since such effects have been observed in the case of the B[*a*]P-*N*^2^-dG adducts (56,71). Differences in the lengths of the DNA substrates used in different laboratories could also contribute to the variations in incision yields reported.

### DNA duplexes are thermally destabilized by 5′,8-cyclopurine-2′-deoxynucleotide lesions and cdA is more destabilizing than cdG

The thermal stabilities of the 17-mer duplexes 5′-d(CCACCAAC**X**CTACCACC)•3′-(GGTGGTTG**Y**GATGGTGG) with X = 5′*S* or 5′*R*–cdG and Y = C, or X = 5′*S* or 5′*R*–cdA and Y = T were obtained by analysis of the UV melting profiles. Typical UV melting profiles are shown in Supplementary Figure S4 and the melting points of the duplexes are summarized in Table 1. All four 5′,8-cyclopurine-2′-deoxynucleotide lesions destabilize the 17-mer duplexes as shown by the differences in the melting points (*T_m_*), Δ*T_m_* = *T_m_* (modified) – *T_m_* (unmodified). The Δ*T_m_* values are near −3 and −5 to 6°C in the case of duplexes with either the cdG or the cdA lesions in the 17-mer duplex sequence context investigated (Table 1). Analogous destabilizations due to 5′,8-cyclopurine-2′-deoxynucleotide lesions with *S* absolute configurations have been observed previously in different sequence contexts and oligomer lengths in the case of the 5′*S–*cdA (51,54) and 5′*S–*cdG (50) cyclopurines.

**Table 1.** Melting points, *T_m_*, of 17-mer DNA duplexes

5′-CCACCAACXCTACCACC	*T_m_*, °C
3′-GGTGGTTGYGATGGTGG	
X = A, Y = T (unmodified)	65.2 ± 0.6
X = 5′*R*–cdA, Y = T	58.9 ± 0.6
X = 5′*S–*cdA, Y = T	60.5 ± 0.6
X = G, Y = C (unmodified)	66.2 ± 0.7
X = 5′*R*–cdG, Y = C	63.4 ± 1.0
X = 5′*S–*cdG, Y = C	63.5 ± 0.6

Our MD simulations (discussed more fully further below) show that one of the cdA:T Watson–Crick hydrogen bonds is very perturbed and dynamic (Supplementary Figure S5), as also observed by NMR methods which revealed fast imino proton solvent exchange for the 5′*S–*cdA:T pair (51); this dynamic perturbation contrasts with the stability of the 5′*S*–cdG:C pairing, also shown in Supplementary Figure S5, and the difference between cdA and cdG is common to both stereoisomers. The local base-pairing instability for the cdA lesions appears to propagate, making it easier for them to melt compared to the cdG lesions. However, both cdA and cdG lesions are locally destabilizing as a result of severe DNA backbone distortions, with a variety of helical perturbations produced in both cases, as seen in our MD simulations detailed below, and in the NMR solution structures (50,51). The differential impact of the 5′*R* and 5′*S* stereoisomers on local dynamics, distortions and base–base stacking interactions, is not reflected in the global melting points of the 17-mer duplexes, but manifests itself in the NER dual incision efficiencies. According to present models, the DNA lesions are recognized by NER proteins via the local thermodynamic destabilization around the lesion site (42,72,73), while the global melting points of DNA duplexes are associated with longer range, cooperative dissociation effects; however, the melting points are affected by the lesions as well, especially in the case of shorter DNA duplexes.

### Modeling and molecular dynamics simulations provide insights into the structural differences between the 5′*R*– and 5′*S*–cdG and cdA lesions

**Figure 4.**
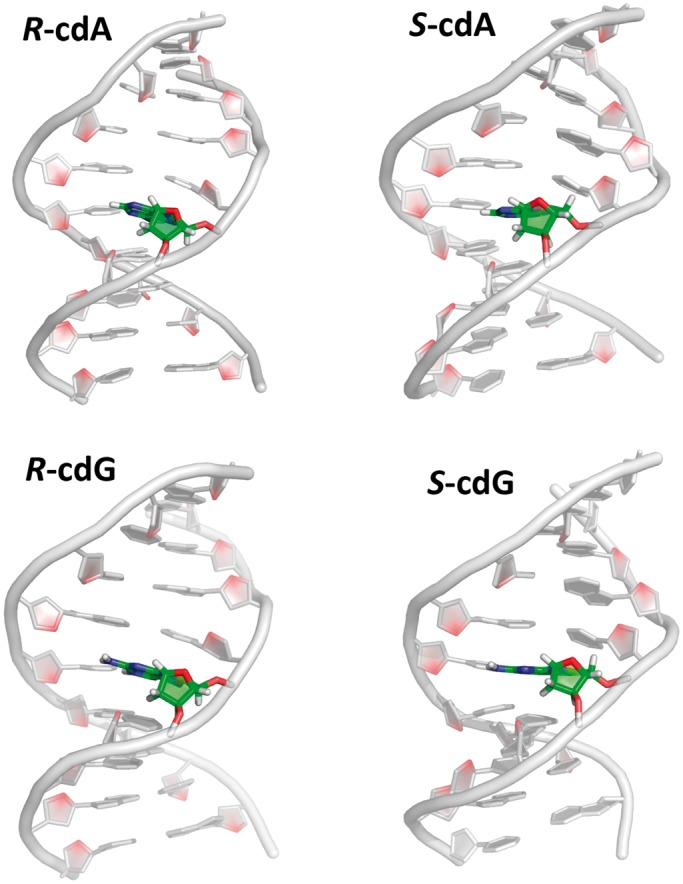
Central 9-mers of the best representative structures from the MD simulations of the 5′*R* and 5′*S*–cdA and cdG-containing 11-mer duplexes. Supplementary Figure S14 shows the corresponding unmodified controls.

**Figure 5.**
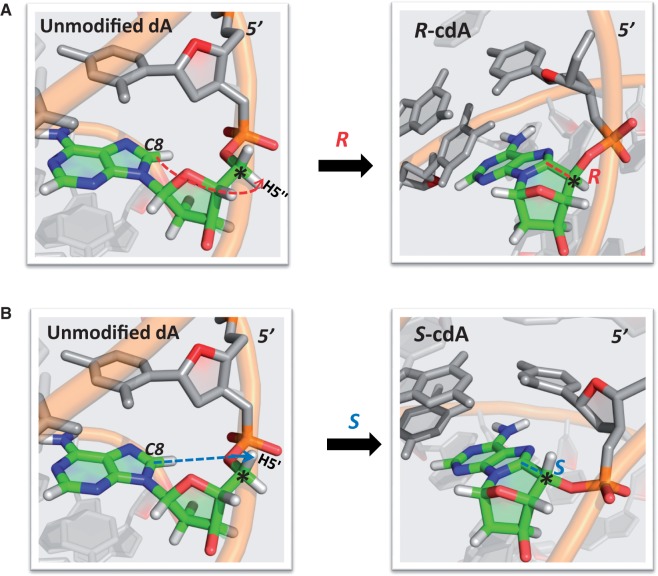
Formation of 5′*R* and 5′*S* stereoisomer cdA lesions from unmodified DNA. The structures utilized are the best representative structures from the MD simulations of the 5′*R*–cdA, and 5′*S*–cdA-containing duplexes, and their corresponding unmodified duplex 11-mers. The lesion site is colored by atom: C, green; N, blue; O, red; H, white. Other bases are grey with O4′ in red, and the backbone is orange in cartoon view. Note the different orientations of the DNA backbones in the 5′*R* and 5′*S* stereoisomers upon formation of the lesions. Movies S6 and S7 show an animated version of this figure. A comparable figure for cdG is given in Supplementary Figure S6.

We have carried out 100 ns of MD simulations for the 5′*R* and 5′*S–*cdA and cdG lesions in 11-mer duplexes as well as control simulations for the two unmodified duplexes as detailed in the Materials and Methods section. The best representative structures from the MD simulations containing the cyclopurine lesions are shown in Figure 4. Movies S1–S4 (Supplementary Material) show an extracted duplex trimer with the cyclopurine lesions in the center, and views are along and perpendicular to the axes of the mini-helices. The key property of the 5′*R* and 5′*S* cyclopurine stereoisomers is the new covalent bond that is formed between the C5′ and the C8 atom on the adenine or guanine base, replacing one or the other hydrogen atom on C5′, namely H5′′ and H5′, respectively. Figure 5 compares the most representative structures from the MD simulations for the unmodified duplex with X = A (Figure 1) on the left in A and B, together with the structures for the 5′*R* and 5′*S* stereoisomers on the right. The figure designates, in the case of the unmodified duplex, the two hydrogen atoms, H5′ and H5′′, that are individually substituted by the single carbon atom (C8) to generate the 5′*R* and 5′*S* stereoisomers, respectively. A comparable illustration for the X = G case is given in Supplementary Figure S6.

#### Unusual O4′-exo sugar puckers and anti glycosidic bond conformations are common structural features of the cyclopurine lesions

The new covalent bond that forms the cyclo-ring in both 5′*R* and 5′*S* stereoisomers forces the sugar pucker into the unusual O4′-exo envelope conformation, with O4′ out of plane; this pucker is energetically disfavored for unmodified DNA because of the crowding between the axial, eclipsed glycosidic and C4′–C5′ bonds (74,75). Supplementary Table S4 shows values of the sugar pucker pseudorotation parameters ***P*** and *τ*_max_ that characterize the sugar puckering and the out of plane position of the O4′ atom, respectively (76). We observed that ***P*** and *τ*_max_ are rigidly constrained to very narrow regions (∼280° and ∼49°, respectively) independent of stereochemistry or the base that is modified. This pucker imposes non-planarity on the cyclo-ring in both stereoisomers, whose most prominent out-of-plane atom involves the locked-in O4′. The glycosidic bond is likewise restrained to the *anti* conformation, but is not rigid due to some flexibility of the cyclo-ring (Supplementary Figure S7). Crystal structures at the damaged base level (adenine) (77,78), NMR solution structures (50,79,80) and computational studies (80–82) have previously noted that these structural properties are essentially independent of stereochemistry and the purine base modified.

#### Formation of the cyclo-ring produces greater backbone dynamics in the case of the 5′R than the 5′S stereoisomer

The NER incision results showed that the *R* stereoisomer is excised more efficiently than the *S* 5′,8-cyclopurine lesions irrespective of the modified base, which is consistent with previous observations reported for the 5′*R* and 5′*S* diastereoisomeric cdA lesions (35). Here we focus on elucidating the structural, dynamic and energetic reasons for this stereoisomer effect.

**Figure 6.**
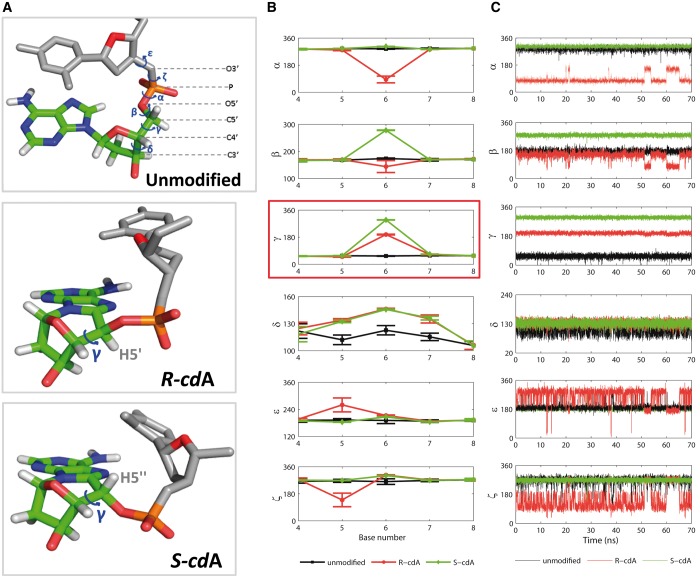
Stereoisomer-dependent impact of 5′*R*–cdA and 5′*S*–cdA lesions on the B-DNA backbone torsion angles (76). (**A**) Backbone torsion angle definitions and best representative structures from the MD simulations of the 5′*R*–cdA, 5′*S*–cdA and the unmodified duplex. (**B**) Block averages and standard deviations of block averages for backbone torsion angles (degrees) of central 5-mers. Note that *δ* which governs the O4′ sugar pucker is stereoisomer-independent, while all other backbone torsions differ in the 5′*R* and 5′*S* stereoisomers. The origin of this difference is in *γ* in the cyclo-ring which is restrained to the *trans* and *gauche^−^* domains in the 5′*R* and 5′*S* stereoisomers, respectively. (**C**) Time dependence of the backbone torsion angles between base cdA/A6 and C5, showing greater dynamics for the 5′*R* stereoisomer. A comparable figure for cdG is given in Supplementary Figure S8.

The formation of the new covalent bond producing the cyclo-ring has a different impact on the DNA backbone in closing the cyclo-ring in the 5′*R* and 5′*S* cases. The backbone torsion angles of the unmodified DNA must rotate differently to produce the 5′*R* and 5′*S* stereoisomers as shown in Figure 6 for the cdA stereoisomers and Supplementary Figure S8 for the cdG case; only the angle *δ* which reflects the restrained O4′-exo sugar pucker remains unchanged for both stereoisomers. The key torsion angle that defines the backbone differences is the C3′–C4′–C5′–O5′ bond *γ* (Figure 6). Because of the formation of the 5′*R* stereoisomer covalent linkage between the C8 and the H5′′ positions of C5′, the *γ* torsion angle must rotate into the *trans* orientation (ensemble average value = 198 ± 2°) from the normal *gauche^+^* domain in B-DNA (ensemble average value = 55 ± 1°) (76). In contrast, in forming the 5′*S* stereoisomer with the covalent bond between C8 and the C5′ H5′ position, *γ* must rotate into the *gauche^−^* domain (ensemble average value = 295 ± 1°). The locking of *γ* in the *trans* and *gauche^−^* orientations in the 5′*R* and 5′*S* stereoisomers, respectively, compared to *gauche^+^* in the unmodified case, causes the subsequent torsion angle changes indicated in Figure 6. Strikingly, the backbone torsion angles, except for the locked sugar pucker and C4′–C5′ bonds, are very dynamic in the 5′*R* but not in the 5′*S* stereoisomers. This is seen from the time-dependence of the torsion angles (Figures 6C for cdA and Supplementary Figure S8C, for cdG). These oscillations occur because most backbone torsions, notably *α*, *ε* and *ζ*, are forced into domains that are more distorted, compared to the unmodified DNA, in the 5′*R* than the 5′*S* stereoisomeric lesions (Figure 6B); hence, the dynamics reveal repeated transient torsion angles excursions in an effort to adopt more favorable conformations but they are forced back by the constrained C4′–C5′ bond.

#### Local helical and base pair distortions differ subtly in the 5′R and 5′S stereoisomers

**Figure 7.**
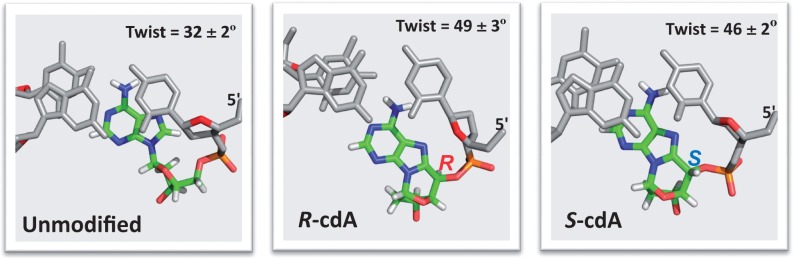
(A) Best representative structures looking down the helix axis of the (C5:G18)-(cdA:T17) base pair step show the overtwisting, compared to its counterpart step in the unmodified case. A comparable figure for cdG is given in Supplementary Figure S9.

The different backbone torsion angles in the two stereoisomeric lesions compared to the unmodified B-DNA cause overtwisting, and the backbone differences between the 5′*R* and 5′*S* stereoisomers lead to modestly greater overtwisting in the 5′*R* case. The 5′*R* lesions are overtwisted between the C5:G18 and cdA/cdG:T/C17 base pairs, relative to the unmodified cases, by ∼17°, while the 5′*S* lesions are overtwisted by 13–14° (Figures 7 and Supplementary Figure S9, and stereoviews of cdG and cdA are shown in Supplementary Figure S10). The local overtwisting has been observed in the NMR solution structures of 5′*S*–cdA and cdG lesions (50,51), but there are no NMR solution structures to date for the 5′*R* stereoisomers. We note that the local overtwisting is compensated by undertwisting in the base pair steps between A4:T19 and C5:G18, and between cdA/cdG:T/C17 and C7:G16 (Supplementary Figure S11 and Table S5) as they adjust in an effort to bring the adjacent B-DNA towards its normal mean twist angle of ∼36° (76).

There are also further local distortions in the helical parameters, such as Roll, Tilt and Shift and perturbations to hydrogen bonding that do not rupture base pairing, that differ in the 5′*R* and 5′*S* cases. Disturbances in local hydrogen bonding (e.g. Propeller and Buckle) at and near the lesion site are mainly confined to the central trimer, and are dampened in base pairs further away from the lesion. These properties are often more dynamic in the 5′*R* than the 5′*S* lesions, which is seen in the higher standard deviations of block averages (computed as detailed in the Materials and Methods section). The full analyses of the helical parameters derived from our ensembles are provided in Supplementary Figure S11 and Table S5 for both cdA and cdG 5′*R* and 5′*S* stereoisomers.

Movie S5, Supplementary Material, illustrates the dynamics of the 5′*R* and 5′*S*–cdA lesions in duplex trimers extracted from the duplex 11-mers, showing the greater dynamics and perturbations of the 5′*R* stereoisomeric lesion. Enhanced dynamics and perturbations for the 5′*R* lesions are associated with their greater NER incision susceptibility.

### *Stacking is more impaired in the* 5′*R than the* 5′*S stereoisomer*

**Figure 8.**
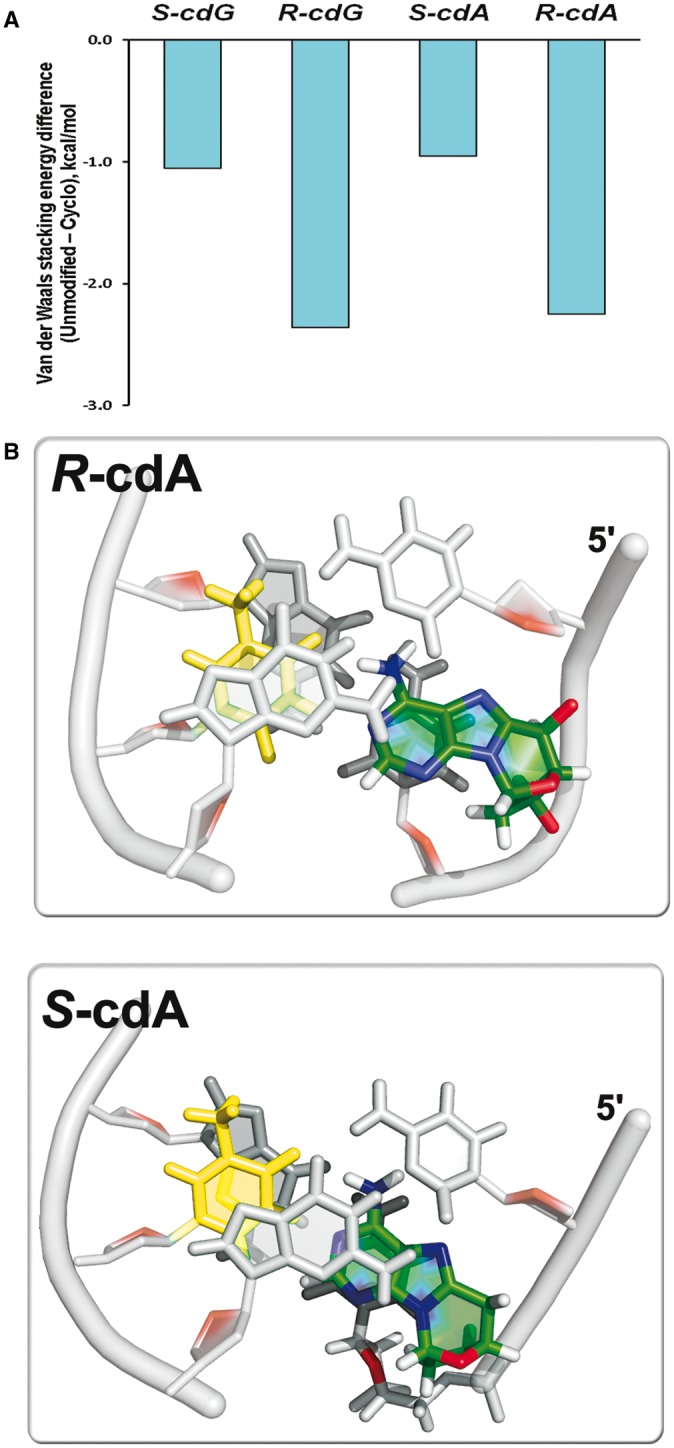
(**A**) Base pair stacking interaction energy differences between the cyclopurine modified and the respective unmodified duplexes (see Materials and Methods section). Supplementary Table S6 gives the stacking energy values and standard deviations of block averages. (**B**) Best representative structures looking down the helix axis of the (C5:G18)-(cdA:T17)-(C7:G16) central trimer of the duplex 11-mer show that the greater backbone distortions in the 5′*R*-stereoisomer lead to diminished stacking on the damaged strand for 5′*R*–cdA, compared to 5′*S*–cdA. The cdA lesions are colored by atom: C, green; N, blue; O, red; H, white. Partner base T17 is light yellow, and the adjacent bases are grey. A comparable figure for cdG is given in Supplementary Figure S13. Movies S1–S4 in Supplementary Material also include views looking down the helix axis.

The distortions produce disturbance to base–base stacking interactions in the central trimer of the duplexes induced by either of the two stereoisomers. However, the enhanced dynamics that are concomitant with the greater backbone distortion, cause diminished base–base stacking interactions that are more pronounced in the 5′*R* stereoisomers. This is seen in the van der Waals interaction energies for the central trimer (Supplementary Table S6). Figure 8A depicts the energies relative to the respective unmodified duplexes, and Supplementary Figure S12 shows that stacking energy is more dynamic in the 5′*R* stereoisomer as a result of its greater backbone dynamics. Figure 8B provides a view of the central trimer for the 5′*S* and 5′*R* cdA lesions looking down the helix axis. In the case of the damaged strand, the notable observation is that the 5′*R*–cdA is essentially unstacked on its 5′-side, while it is stacked on its 3′-side with C7 in both stereoisomers. However, stacking in the partner strand is approximately equivalent in the 5′*R* and 5′*S* stereoisomers: the central T17 stacks with its 5′-side base G18 and its 3′-side base G16 and the extent of stacking is nearly the same in the two cases. Hence, stacking is less in the modified strand for the 5′*R* than the 5′*S* case. These strand-dependent stacking differences are evident from the van der Waals interaction energies presented in Supplementary Table S6. Stacking phenomena are similar for the cdG stereoisomer pair (Supplementary Figure S13 and Table S6).

#### NER incision efficiencies correlate with greater stacking impairment in the case of the 5′R lesions

The stereoisomer-dependent differences in DNA backbone conformational perturbations and dynamics with concomitant helical distortions, produce stacking differences that correlate with the observed stereoisomer-dependent NER differences (Figure 3). The correlation between NER incision efficiencies and stacking interactions, is in line with our earlier hypothesis that van der Waals stacking interaction effects contribute to our understanding of relative NER recognition and incision efficiencies. We have found that lesions that provide enhanced stacking interactions are less NER-susceptible, while diminished stacking interactions are correlated with enhanced NER (83,84). The crystal structure of Rad4/Rad23, the yeast homolog of the human NER lesion recognition factor XPC/RAD23B reveals that the lesion-partner base is flipped out of the helix and binds to amino acids of the protein. The Rad4 BHD3 β-hairpin intrudes between the damaged duplex at the lesion site for recognition, with concomitant flipping of two partner bases into the protein (72). Both β-hairpin intrusion and flipping are facilitated by local thermodynamic destabilization that includes weakened stacking interactions (73,85).

## CONCLUSIONS

We have determined for the first time the relative NER efficiencies of 5′*R* and 5′*S* cdA and cdG lesions in the identical sequence contexts and in the same HeLa cell extract preparations, in order to afford a direct comparison of relative NER incision efficiencies of the four 5′,8-cyclopurine-2′-deoxynucleotide lesions. Such direct comparisons are needed to determine the mechanistic basis of the differential recognition and excision of the two pairs of stereoisomeric lesions. On the basis of these results, we conclude that the 5′*R*–cdA and 5′*R*–cdG lesions are better NER substrates than the 5′*S*–cdA and 5′*S*–cdG lesions. Furthermore, the differences in NER cleavage efficiencies between cdA and cdG lesions with the same absolute configurations embedded in our sequence context are more minor. These NER results are consistent with the differences in lesion-induced distortions and dynamics, and their impact on local base stacking interactions. These effects are revealed by the molecular dynamic simulation studies: both the 5′*R*–cdA and 5′*R*–cdG stereoisomeric lesions are significantly more dynamic and more distorting than the respective 5′*S* stereoisomers and manifest a greater local duplex destabilization with diminished base–base stacking interactions. However, the cdA and cdG lesions that have the same absolute configurations exhibit similar properties and stacking interactions. The greater stacking impairment in the 5′*R* stereoisomer correlates well with its greater relative NER excision efficiency. While these structural differences appear to play a role in the recognition of the 5′,8-cyclopurine-2′-deoxynucleotide lesions in DNA duplexes by human NER-recognition factors, it will be interesting to learn how the distortions impact the susceptibility of these same lesions in nucleosomes where NER is generally severely inhibited (86–88).

## SUPPLEMENTARY DATA

Supplementary Data are available at NAR Online.

Supplementary Data
