# Supplementary material for: Structural basis for the recognition of diastereomeric 5′,8-cyclo-2′-deoxypurine lesions by the human nucleotide excision repair system
Source: Nucleic Acids Res. 2014 Mar 10;42(8):5020–32. doi: 10.1093/nar/gku162 (PMC4041128; doi:10.1093/nar/gku162)
Supplement: Supplementary Data [file supp_42_8_5020__index.html]

Structural basis for the recognition of diastereomeric 5′,8-cyclo-2′-deoxypurine lesions by the human nucleotide excision repair system — Supplementary Data 

# Structural basis for the recognition of diastereomeric 5′,8-cyclo-2′-deoxypurine lesions by the human nucleotide excision repair system

## Supplementary Data

files

**Files in this Data Supplement:**

- Supplementary Data - pdf file
- Supplementary Data - avi file
- Supplementary Data - avi file
- Supplementary Data - avi file
- Supplementary Data - avi file
- Supplementary Data - avi file
- Supplementary Data - avi file
- Supplementary Data - avi file
