# Supplementary material for: Structural basis for the recognition of diastereomeric 5′,8-cyclo-2′-deoxypurine lesions by the human nucleotide excision repair system
Source: Nucleic Acids Res. 2014 Mar 10;42(8):5020–32. doi: 10.1093/nar/gku162 (PMC4041128; doi:10.1093/nar/gku162)
Supplement: Supplementary Data [file supp_gku162_cyclo_supporting_revision.pdf]

Supplementary Data

Structural basis for the recognition of diastereomeric 5',8-cyclo-2'-deoxypurine lesions by the human nucleotide excision repair system

Konstantin Kropachev[1], Shuang Ding[2], Michael A Terzidis[3], Annalisa Masi[3], Zhi Liu[1], Yuqin Cai[2], Marina Kolbanovskiy[1], Chryssostomos Chatgilialoglu[3], Suse Broyde[2,*], Nicholas E Geacintov[1,*], Vladimir Shafirovich[1,*]

[1] Department of Chemistry and [2] Biology, New York University, 100 Washington Square East, New York, NY 10003, United States, and [3] Istituto per la Sintesi Organica e la Fotoreattività, Consiglio Nazionale delle Ricerche, Via P. Gobetti 101, 40129 Bologna, Italy

*To whom correspondence should be addressed. Tel: +1 212 998 8456; Fax: +1 212 998 8421; Email: vs5@nyu.edu. Correspondence may also be addressed to Nicholas E. Geacintov, Tel: +1 212 998 8407; Fax: +1 212 998 8421; Email: ng1@nyu.edu, or Suse Broyde Tel: +1 212 998 8231; Fax: +1 212 995 4015; Email: broyde@nyu.edu

Table of Contents

Experimental supplementary data……………………………………………………….3-5

Figure S1. Base sequences of 147-mer oligo-2'-deoxyribonucleotide sequences.

Figure S3. Comparison of NER kinetics determined in parallel experiments with ODN(1) and ODN(2) duplexes.

Figure S4. Examples of UV melting curves of 17-mer duplexes.

Molecular modeling and MD simulation supplementary data……………………… 6-27

MD simulation protocol and block average calculations

Table S1. Added force field parameters for the cyclopurines.

Tables S2 and S3. AMBER atom type, connection type, and partial charges for the cyclopurine lesions

Table S4. Sugar pucker pseudorotation parameters ***P*** and τ_{max} values.

Table S5. Block averages and standard deviations of helical parameters.

Table S6. Van der Waals interaction energies involving the lesion site.

Figure S2. RMSD vs time plot for the MD simulations.

Figure S5. Hydrogen bond distance at the lesion site vs time plot for the 5'*S*-cdA and cdG lesions.

Figure S6. Formation of 5'*R* and 5'*S* stereoisomeric cdG lesions from unmodified DNA.

Figure S7. Glycosidic torsion angle of the lesion site vs time plot for the MD simulations.

Figure S8. Stereoisomer-dependent impact of 5'*R*- and 5'*S*-cdG lesions on the B-DNA backbone torsion angles.

Figure S9. Dimers of cdG-modified duplex structures looking down the helix axis and ensemble average Twist difference.

Figure S10. Stereo views of dimers looking down the helix axis show the overtwisting.

Figure S11. Ensemble average values of base pair parameters and standard deviations for the cyclo-modified duplexes and unmodified controls.

Figure S12. Time-dependent van der Waals interaction energies of the central trimer.

Figure S13. Central trimers of cdG-modified duplexes looking down the helix axis

Figure S14. Best representative structures of the unmodified duplexes.

References

1. ODN(1). X is the 66th nucleotide counted from the 5'-end.

5'-CACAGGATGT ATATATCTGA CACGTGCCTG GAGACTAGGG AGTAATCCCC (1 - 50)
TTGGCGG**CCA CCAACXCTAC CACC**GCGCGT ACGTGCGTTT AAGCGGTGCT (51 – 100)
AGAGCTGTCT ACGACCAATT GAGCGGCCTC GGCACCGGGA TTCTCCA -3' (101 – 147)

2. ODN(2). X is the 70th nucleotide counted from the 5'-end.

5'-CACAGGATGT ATATATCTGA CACGTGCCTG GAGACTAGGG AGTAATCCCC (1 - 50)
TTGGCGGTTA A**CCACCAACX CTACCACC**GT ACGTGCGTTT AAGCGGTGCT (51 – 100)
AGAGCTGTCT ACGACCAATT GAGCGGCCTC GGCACCGGGA TTCTCCA -3' (101 – 147)

Figure S1. Base sequences of 147-mer oligo-2'-deoxyribonucleotide sequences ODN(1) and ODN(2) with X = A or G (unmodified duplexes), or X = cdA or cdG. These sequences were annealed with their individual complementary partner strands (not shown) with T opposite X = A or cdA, or C opposite G or cdG, to form the ODN(1) and ODN(2) duplexes. The 17-mer sequence (Fig. 1, main text) is shown in red.

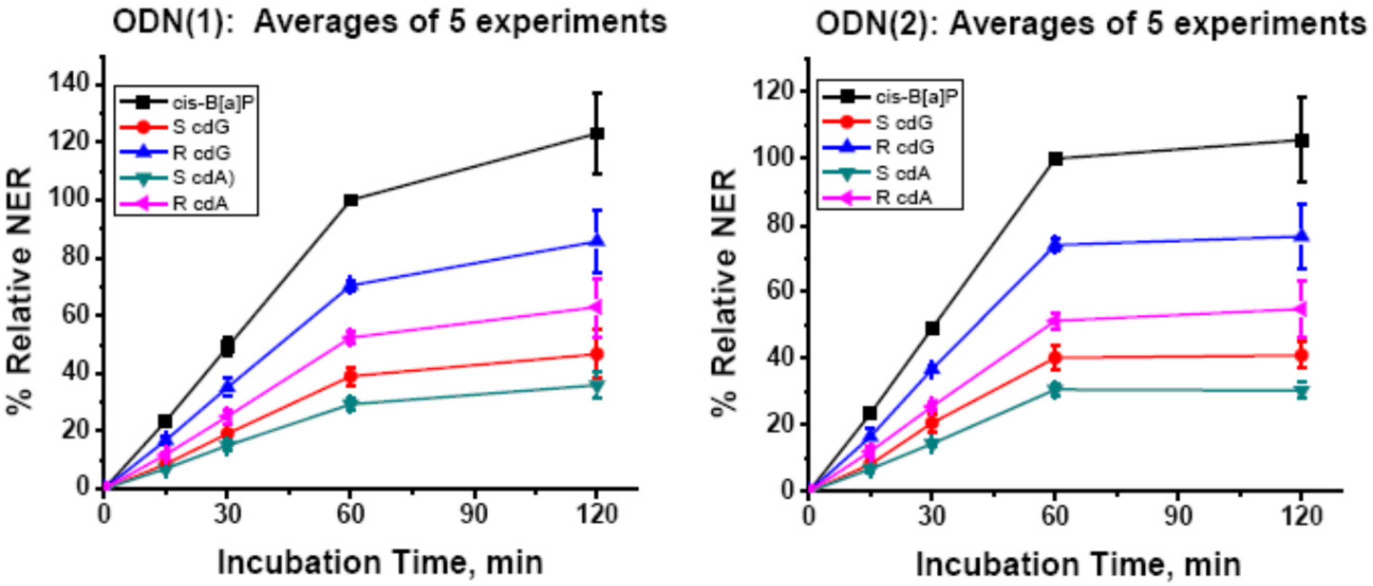

Figure S3. Comparison of NER kinetics determined in parallel experiments with ODN(1) and ODN(2) duplexes. The averages of the slopes of both sets of experiments are shown in the bar graph (Figure 3B, main text). The ODN(1) panel above is the same as in Figure 3A, and the ODN(2) results are shown in the panel on the right. Each panel depicts the averages of five independent experiments performed with different cell extracts. The error bars represent the standard deviations.

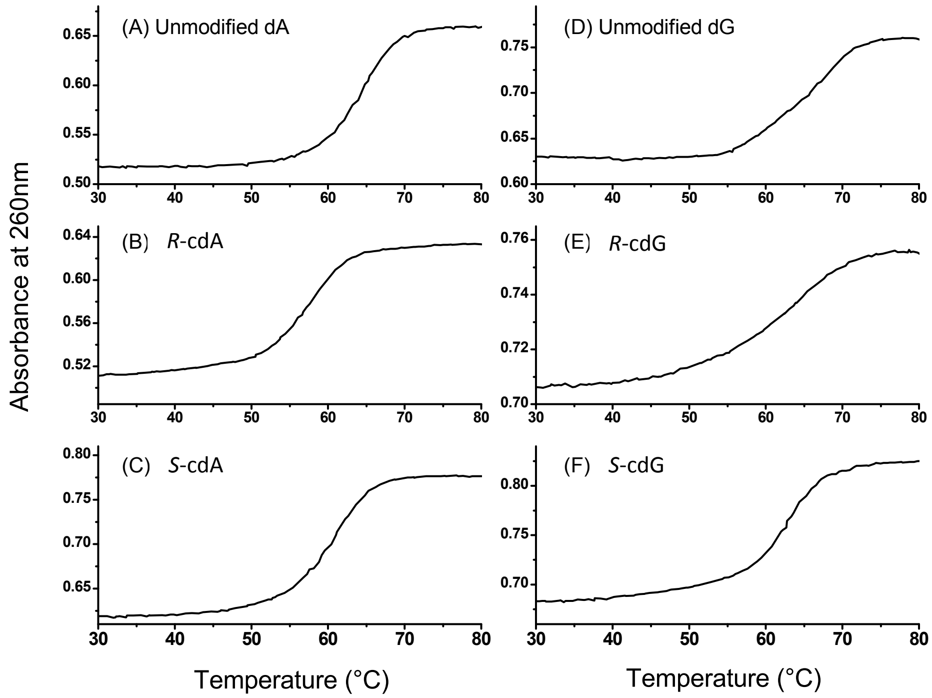

Figure S4. Examples of UV melting curves of 17-mer duplexes (the sequences of the modified and unmodified oligonucleotides are identified in Figure 1 of the main text and Figure S1, shown in red) with the central base identified in each panel.

MD simulation protocol

The duplex starting model was neutralized with 20 Na^+ ions and solvated with explicit water using the LEAP module of AMBER (57). A periodic rectangular box of TIP3P (85) water with 10.0Å buffer was created around the DNA. The particle-mesh Ewald (PME) method (86, 87) with a 9.0 Å cutoff for the nonbonded interactions was used in the subsequent energy minimizations and dynamic simulations. Initially, 500 steps of steepest descent minimization followed by 500 steps of conjugate gradient minimization were conducted for the waters and sodium ions with 500 kcal/(mol·$Å^2$) restraints placed on the DNA duplex. Then, 1000 steps of steepest descent minimization followed by 1500 steps of conjugate gradient minimization were carried out for the entire system without restraints. A 2 fs time step and the SHAKE algorithm (88) were applied in the MD simulations. The system was heated from 0 K to 300 K over 20 ps with a weak 10 kcal/(mol$Å^2$) restraint on the DNA at constant volume. The equilibration was continued with further 100 ps of simulation without restraint at constant atmospheric pressure. Finally, production was carried out for 100 ns at 300 K with a 4.0 ps heat bath coupling parameter.

Block averages and standard deviations of block averages

Block averages and standard deviations of block averages were computed as follows: The raw time series of data produced by the MD simulations are series of correlated data. In order to obtain averages and the variances of the averages for these data, we applied the block averaging method (64). In brief, the time series data were divided into "blocks" with a block size that exceeds the longest correlation time. The average for each block was computed and named "block average". The mean and the standard deviation of the block averages were used to represent the average and the variance of averages.

The block size was determined using the convergence of the standard deviation of the block averages (65). For all raw time series data, we computed the standard deviation of the block averages using a block size from 0.5 ns to 30 ns with an increment of 0.5 ns. The standard deviation values were plotted against the block sizes. The optimal block size was chosen at the point where the standard deviation values converge, reaching a plateau (data not shown). For our 70 ns time series data, the block size was chosen at 1.4 or 3.5 ns, resulting in 50 or 20 blocks for each time series data.

Table S1. Added force field parameters for the cyclopurines.

bond	K_r (kcal/(mol Å^2))	r_{eq} (Å)
N*-CC	436	1.36

angle	K_θ (kcal/(mol radian^2))	θ_{eq} (deg)
CC-N*-CB	70	106.80
CC-NB-CB	70	103.80
N*-CC-NB	70	113.91
CT-N*-CC	70	124.41
H1-CT-CC	50	105.33
CT-CC-N*	70	116.70
OS-CT-CC	50	114.95

torsion	no. of paths	$V_n/2$ (kcal/mol)	γ (deg)	n
X -CC-N*-X	4	6.6	180	2

Table S2. AMBER atom type, connection type, and partial charge assignments for the cdA adducts.

Atom name	Atom type	Connection type	Partial charge	
			5'*S*-cdA	5'*R*-cdA
P	P	M	1.221956	1.221476
O1P	O2	E	-0.793000	-0.793400
O2P	O2	E	-0.793000	-0.793400
O5'	OS	M	-0.506407	-0.495400
C5'	CT	M	0.037045	0.049311
H5'	H1	E	0.165219	0.154804
C4'	CT	M	0.058725	0.043819
H4'	H1	E	0.168319	0.162246
O4'	OS	S	-0.419900	-0.355200
C1'	CT	B	0.166995	0.064158
H1'	H2	E	0.155242	0.148479
N9	N*	S	-0.072421	-0.033906
C8	CC	S	0.299678	0.322020
N7	NB	S	-0.596807	-0.587100
C5	CB	S	0.006836	-0.007731
C6	CA	B	0.716749	0.745196
N6	N2	B	-0.912800	-0.944100
H61	H	E	0.417754	0.418706
H62	H	E	0.417754	0.418706
N1	NC	S	-0.779500	-0.773100
C2	CQ	B	0.605810	0.593865
H2	H5	E	0.054444	0.055380
N3	NC	S	-0.767100	-0.749200
C4	CB	E	0.417076	0.398202
C3'	CT	M	0.104086	0.136931
H3'	H1	E	0.085198	0.044789
C2'	CT	B	-0.014400	-0.033900
H2'1	HC	E	0.039623	0.056652
H2'2	HC	E	0.039623	0.056652
O3'	OS	M	-0.522837	-0.524900

Table S3. AMBER atom type, connection type, and partial charge assignments for the cdG adducts.

Atom name	Atom type	Connection type	Partial charge	
			5'*S*-cdG	5'*R*-cdG
P	P	M	1.220985	1.221882
O1P	O2	E	-0.792233	-0.793200
O2P	O2	E	-0.792233	-0.793200
O5'	OS	M	-0.501580	-0.504005
C5'	CT	M	0.024205	0.091156
H5'	H1	E	0.110977	0.074630
C4'	CT	M	0.047310	0.170009
H4'	H1	E	0.132188	0.132225
O4'	OS	S	-0.402916	-0.378357
C1'	CT	B	0.092393	0.118724
H1'	H2	E	0.154347	0.123816
N9	N*	S	-0.054159	0.079937
C8	CC	S	0.351957	0.226874
N7	NB	S	-0.500043	-0.564176
C5	CB	S	0.009467	0.104621
C6	C	B	0.576649	0.578753
O6	O	E	-0.579260	-0.552740
N1	NA	B	-0.525575	-0.584510
H1	H	E	0.342161	0.366843
C2	CA	B	0.845412	0.792262
N2	N2	B	-0.986549	-0.928000
H21	H	E	0.442075	0.404051
H22	H	E	0.442075	0.404051
N3	NC	S	-0.700348	-0.638500
C4	CB	E	0.224464	0.146985
C3'	CT	M	0.089596	0.070754
H3'	H1	E	0.125129	0.069413
C2'	CT	B	0.058911	-0.084902
H2'1	HC	E	0.043022	0.083859
H2'2	HC	E	0.043022	0.083859
O3'	OS	M	-0.540268	-0.523111

Table S4. Sugar pucker pseudorotation parameters ***P*** and τ_{max} values.[a]

(degrees)	5'*S*-cdA	5'*R*-cdA	Unmodified dA	5'*S*-cdG	5'*R*-cdG	Unmodified dG
P	281 (± 1)	280 (± 1)	135 (± 8)	280 (± 1)	280 (± 1)	145 (± 8)
τ_{max}	48 (± 0.2)	49 (± 0.2)	38 (± 1)	48 (± 0.2)	49 (± 0.3)	38 (± 1)

[a] The mean values and standard deviations of the block averages are given.

Table S5. Block averages and standard deviations of block averages of helical parameters.

		5'*S*-cdA		5'*R*-cdA		Unmodified dA		5'*S*-cdG		5'*R*-cdG		Unmodified dG	
Buckle (°)		avg	std	avg	std	avg	std	avg	std	avg	std	avg	std
	3	3.6	3.7	-1.8	3.6	3.6	4.0	8.6	3.8	1.0	3.9	3.9	5.4
	4	9.1	2.0	2.7	4.4	7.1	2.9	12.7	2.4	3.6	3.6	11.2	4.5
	5	17.4	2.4	16.9	4.0	7.1	2.9	20.0	2.5	16.4	3.1	9.7	4.4
	6	-13.4	3.1	3.7	5.2	1.5	2.9	-13.3	2.8	-1.5	4.3	-0.1	3.1
	7	10.1	1.8	10.5	2.7	1.5	2.3	12.0	1.8	8.7	2.8	-4.3	2.8
	8	5.0	2.7	-0.7	3.8	-2.5	3.6	1.0	2.9	2.4	3.3	-5.5	4.7
	9	-1.1	5.4	-3.3	7.3	-0.3	4.3	-7.5	3.1	-1.0	5.0	1.0	4.2
Propeller (°)		avg	std	avg	std	avg	std	avg	std	avg	std	avg	std
	3	-9.8	2.0	-11.4	3.7	-11.0	2.4	-7.0	2.6	-11.7	2.7	2.3	9.3
	4	-11.8	2.6	-9.9	2.9	-14.3	2.3	-13.1	2.7	-11.9	2.6	-15.0	2.2
	5	-10.4	2.6	0.6	5.7	-12.8	2.3	-12.8	2.3	-3.9	4.1	-14.9	2.3
	6	-6.8	2.9	-23.5	5.4	-12.5	1.8	-5.2	2.6	-17.8	4.4	-11.6	2.3
	7	-2.5	1.2	-8.6	2.4	-10.9	2.4	-6.3	1.5	-6.7	2.4	-10.1	2.4
	8	-16.2	2.8	-16.4	5.1	-13.9	2.3	-22.2	2.5	-15.2	2.6	-12.2	2.5
	9	-11.8	2.9	-10.1	3.0	-11.2	2.3	-11.0	2.9	-9.8	3.4	-8.7	3.8
Opening (°)		avg	std	avg	std	avg	std	avg	std	avg	std	avg	std
	3	2.4	0.7	3.8	0.8	2.7	0.6	1.5	1.1	3.4	1.0	3.3	1.6
	4	2.4	0.7	4.2	1.4	2.8	1.0	1.4	1.3	3.8	1.0	2.3	1.7
	5	1.0	0.7	-0.4	0.8	1.6	0.5	-1.4	2.3	-0.4	0.9	2.4	1.6
	6	11.0	1.0	7.3	1.7	2.7	0.9	7.6	1.5	2.5	0.8	0.8	0.8
	7	-0.9	0.6	0.3	0.9	1.4	0.4	-4.5	1.7	0.0	0.8	1.3	0.7
	8	4.2	1.0	5.1	1.3	4.7	1.1	6.6	2.0	4.4	1.2	4.8	2.2
	9	4.1	1.5	4.1	2.6	3.4	1.4	5.7	3.9	4.0	1.1	2.1	2.6
Rise (Å)		avg	std	avg	std	avg	std	avg	std	avg	std	avg	std
	3	3.2	0.1	3.2	0.1	3.2	0.1	3.4	0.2	3.3	0.1	3.4	0.3
	4	3.1	0.1	3.1	0.1	3.3	0.1	3.4	0.2	3.1	0.1	3.4	0.3
	5	3.9	0.1	3.7	0.1	3.3	0.1	4.3	0.2	3.7	0.1	3.6	0.2
	6	2.8	0.1	3.2	0.2	3.3	0.1	3.1	0.1	3.1	0.3	3.5	0.2
	7	3.4	0.1	3.7	0.1	3.4	0.1	3.6	0.3	3.6	0.2	3.5	0.1
	8	3.3	0.1	3.3	0.1	3.1	0.1	3.6	0.5	3.5	0.2	3.2	0.2

		5'*S*-cdA		5'*R*-cdA		Unmodified dA		5'*S*-cdG		5'*R*-cdG		Unmodified dG	
Roll (°)		avg	std	avg	std	avg	std	avg	std	avg	std	avg	std
	3	-1.1	1.8	2.7	2.5	-1.9	1.9	1.4	3.8	1.0	3.3	-5.9	7.6
	4	0.5	1.3	3.3	2.9	-0.5	1.4	4.8	2.2	3.4	2.6	-1.4	5.1
	5	6.0	1.5	-2.8	4.9	10.1	1.5	12.6	3.3	-1.5	4.8	10.6	3.8
	6	7.1	1.0	8.7	1.1	0.3	1.9	9.9	2.0	8.6	2.3	1.6	2.5
	7	0.7	1.1	1.9	1.6	2.5	1.4	4.3	3.5	3.5	2.3	3.6	2.2
	8	9.2	2.5	9.7	4.5	9.2	2.1	6.0	7.1	12.2	3.0	9.0	4.5
Tilt (°)		avg	std	avg	std	avg	std	avg	std	avg	std	avg	std
	3	-1.1	0.8	-1.7	1.1	-2.7	0.9	0.4	3.4	-1.1	1.7	-4.5	6.9
	4	4.4	0.7	5.5	1.7	-0.3	0.7	6.6	2.9	5.8	1.9	-0.5	5.5
	5	-4.3	1.4	-3.9	2.4	0.2	0.9	1.9	4.8	-5.3	2.7	-1.1	5.6
	6	4.3	1.1	3.2	2.0	-1.2	0.6	8.5	2.5	4.4	2.0	0.4	5.1
	7	2.6	1.8	1.7	2.1	2.6	1.1	11.7	4.3	1.2	2.7	4.4	4.3
	8	2.2	1.1	1.0	2.3	1.5	0.9	13.8	6.3	1.5	2.3	3.1	3.8
Twist (°)		avg	std	avg	std	avg	std	avg	std	avg	std	avg	std
	3	33.5	2.5	32.7	1.8	33.9	2.6	33.9	3.1	34.2	2.5	33.3	2.6
	4	28.7	0.5	23.3	3.4	31.7	1.1	27.8	1.7	24.0	2.9	29.6	2.1
	5	46.4	1.9	49.2	2.9	32.3	1.7	46.8	2.4	51.1	2.1	33.8	2.9
	6	23.3	1.8	29.1	2.1	31.1	1.2	19.9	2.1	27.9	2.2	31.9	1.3
	7	30.5	0.7	30.7	3.1	32.5	1.9	28.8	1.7	31.3	1.9	31.7	2.8
	8	32.4	1.9	31.6	3.3	30.7	1.7	31.2	3.1	30.9	1.7	29.2	3.6
Shift (Å)		avg	std	avg	std	avg	std	avg	std	avg	std	avg	std
	3	0.0	0.1	0.0	0.2	-0.1	0.1	-1.5	0.7	-0.2	0.4	-0.1	1.0
	4	0.1	0.2	0.1	0.3	0.0	0.2	0.5	0.5	0.3	0.3	-0.5	0.8
	5	0.0	0.2	0.1	0.2	-0.1	0.3	0.0	0.3	0.0	0.2	-0.9	0.5
	6	0.5	0.1	0.8	0.3	0.1	0.2	0.4	0.2	0.7	0.3	0.2	0.3
	7	-0.7	0.2	-0.9	0.2	-0.1	0.2	-0.9	0.3	-1.1	0.2	-0.4	0.3
	8	-0.1	0.2	0.1	0.2	0.0	0.2	0.2	0.2	0.1	0.2	-0.1	0.2

Table S6. Van der Waals interaction energies involving the lesion site.

Base pair van der Waals interaction energies (kcal/mol) in central trimer

5'*S*-cdG	5'*R*-cdG	Unmodified dG	5'*S*-cdA	5'*R*-cdA	Unmodified dA
-29.1 (± 0.1)	-27.8 (± 0.4)	-30.2 (± 0.2)	-29.9 (± 0.2)	-28.6 (± 0.3)	-30.8 (± 0.2)

Van der Waals interaction energies (kcal/mol) between bases in central trimer[a]

		5'*S*-cdA	5'*R*-cdA
cdA	C5	-3.5	-3.4
	G18	-4.9	-2.8
	C7	-5.0	-4.9
	G16	-2.2	-1.7
T17	C5	-0.6	-1.3
	G18	-5.5	-6.0
	C7	-0.5	-0.8
	G16	-6.8	-6.9

		5'*S*-cdG	5'*R*-cdG
cdG	C5	-3.5	-3.3
	G18	-5.3	-3.7
	C7	-5.6	-5.6
	G16	-2.7	-2.1
C17	C5	-0.7	-1.3
	G18	-5.3	-5.6
	C7	-0.5	-0.7
	G16	-6.2	-6.3

[a] Highlights show stacking interaction energies that differ most in *R* and *S* stereoisomers.

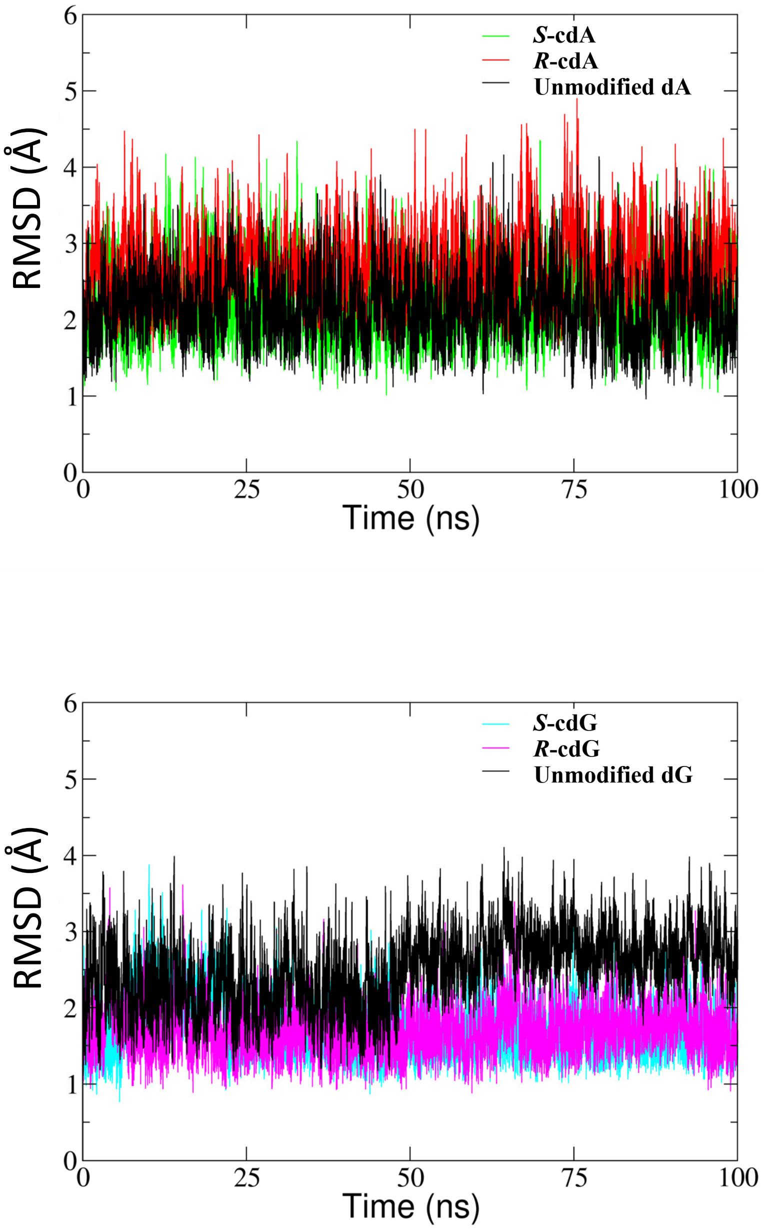

Figure S2. RMSD vs time plot for the MD simulations.

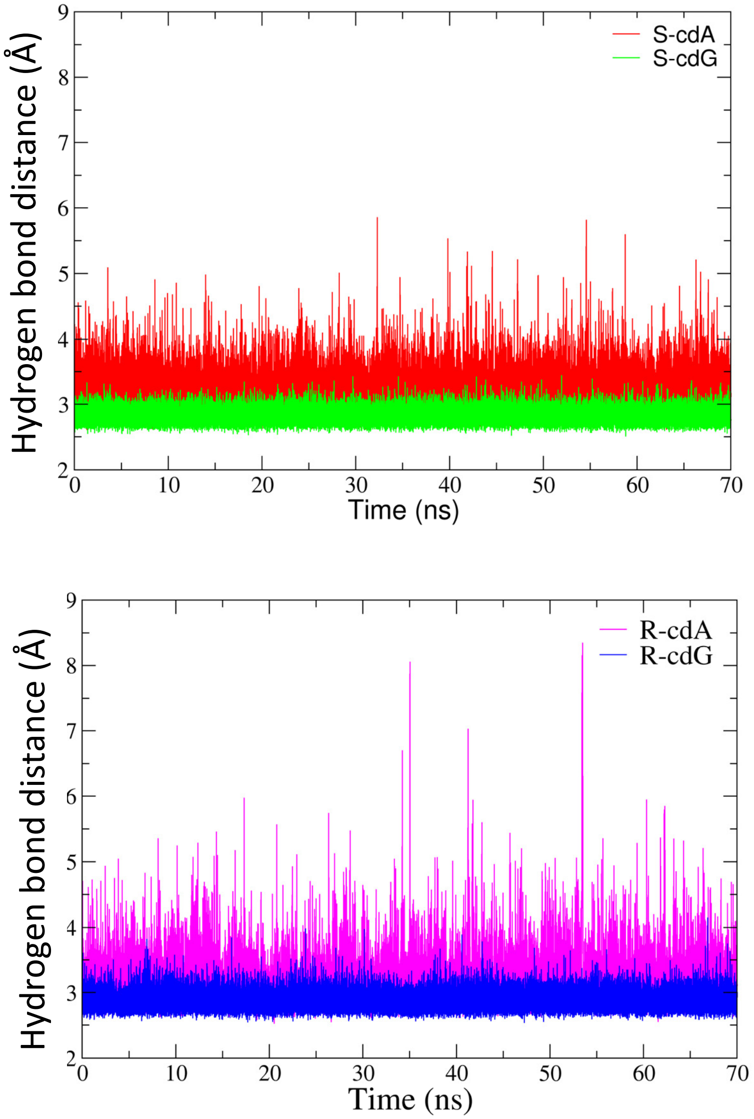

Figure S5. Hydrogen bond distance (heavy atom to heavy atom) at the lesion site vs time plot for the cdA and cdG cyclopurines. In each case, the most disturbed hydrogen bond was selected: cdA(N6) – T17 (O4) and cdG(N2) – C17(O2).

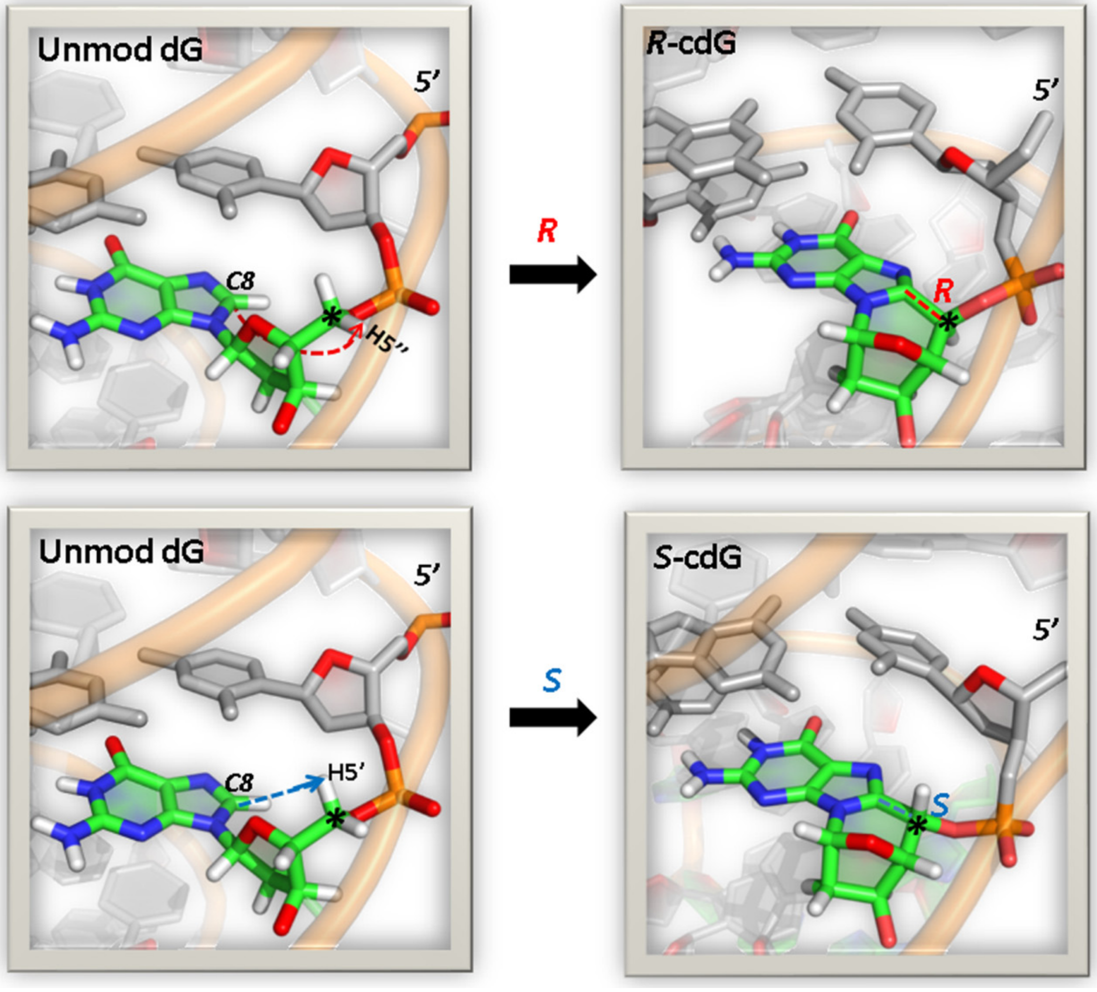

Figure S6. Formation of 5'*R* and 5'*S* stereoisomeric cdG lesions from unmodified DNA. The structures utilized are the most representative structures from the MD simulations of the 5'*R*-cdG, and 5'*S*-cdG containing duplexes, and their corresponding unmodified duplex 11-mer. Note the different orientations of the DNA backbones in the *R* and *S* stereoisomers upon formation of the lesions.

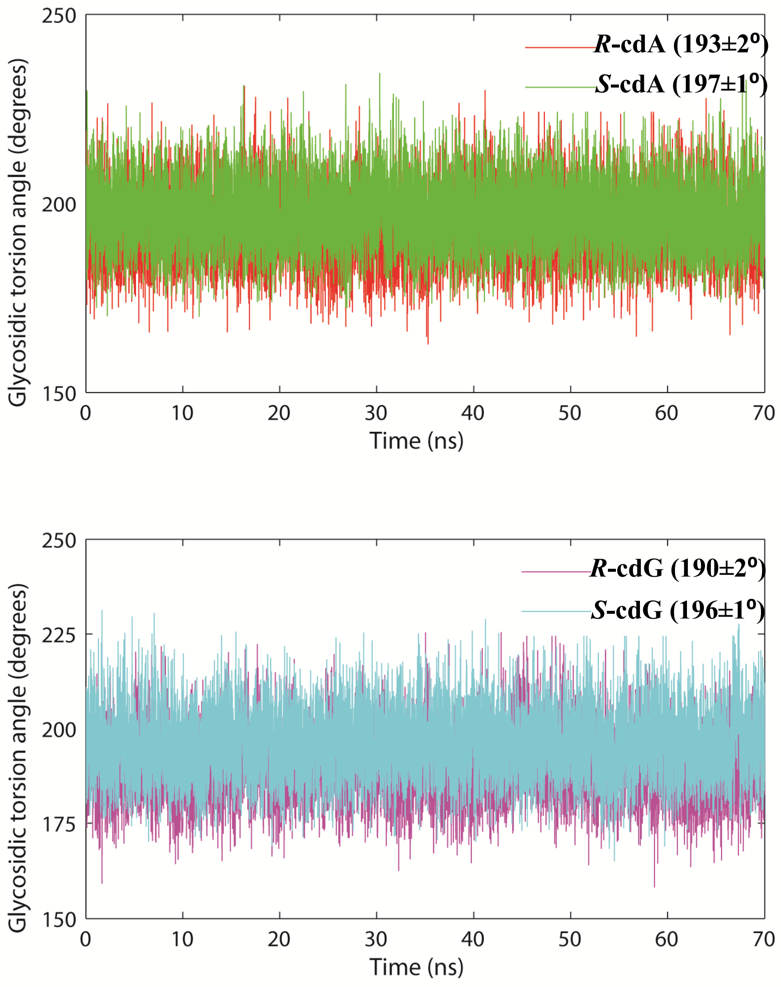

Figure S7. Glycosidic torsion angle of the lesion site vs time plot for the MD simulations. Means and standard deviations of block averages are given.

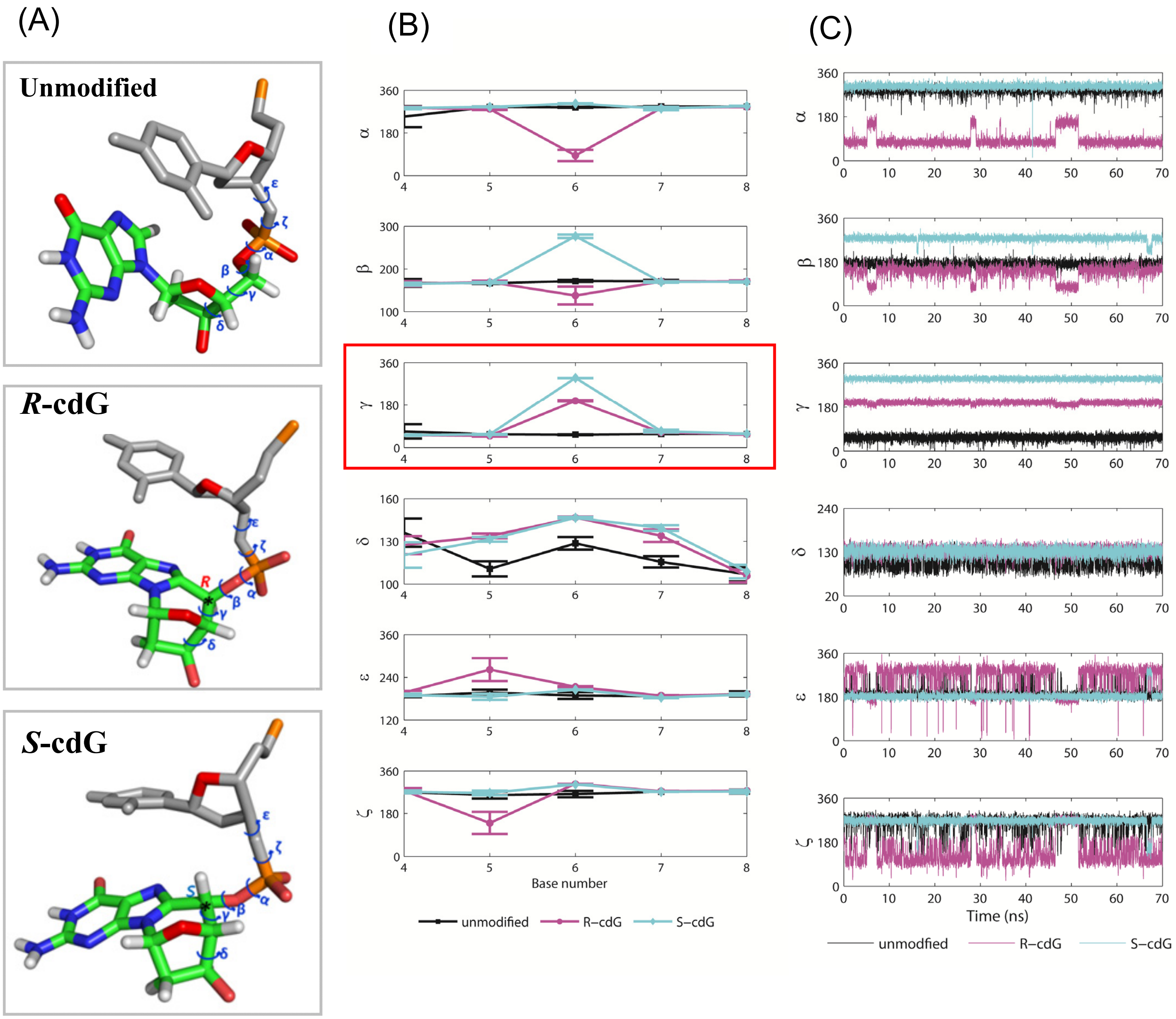

Figure S8. Stereoisomer-dependent impact of 5'*R*-cdG and 5'*S*-cdG lesions on the B-DNA backbone torsion angles. (A) Backbone torsion angle definitions and best representative structures from the MD simulations of the 5'*R*-cdG, 5'*S*-cdG and the unmodified duplex. (B) Block averages and standard deviations of block averages for backbone torsion angles of central 5-mers. Note that δ which governs the O4' sugar pucker is stereoisomer-independent, while all other backbone torsions differ in the 5'*R* and 5'*S* stereoisomers. (C) Time dependence of the backbone torsion angles between base cdG/G6 and C5, showing greater dynamics for the 5'*R* stereoisomer.

(A)

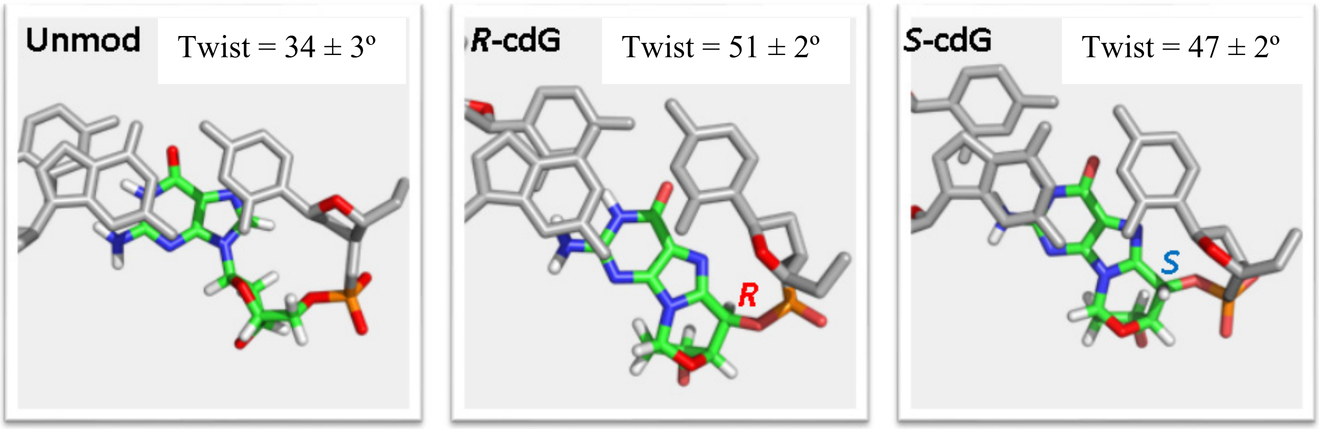

(B)

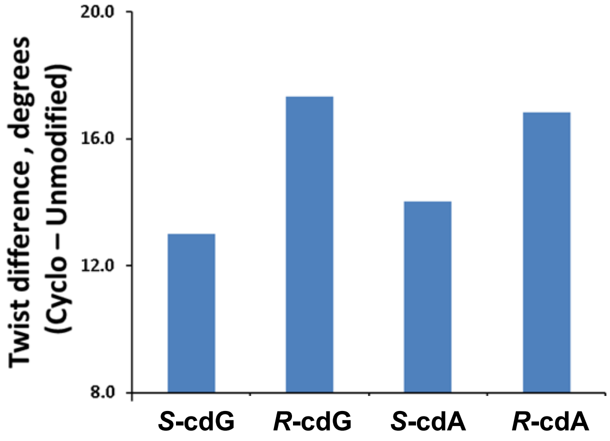

Figure S9. (A) Best representative structures looking down the helix axis of the (C5:G18)-(cdG:T17) base step shows the overtwisting, compared to its counterpart step in the unmodified case. (B) Ensemble average Twist difference between the 5'*R*-cdG, and 5'*S*-cdG, 5'*R*-cdA, 5'*S*-cdA and their respective unmodified control simulation values. Table S5 and Figure S11 give ensemble average values for all helical parameters.

(A)

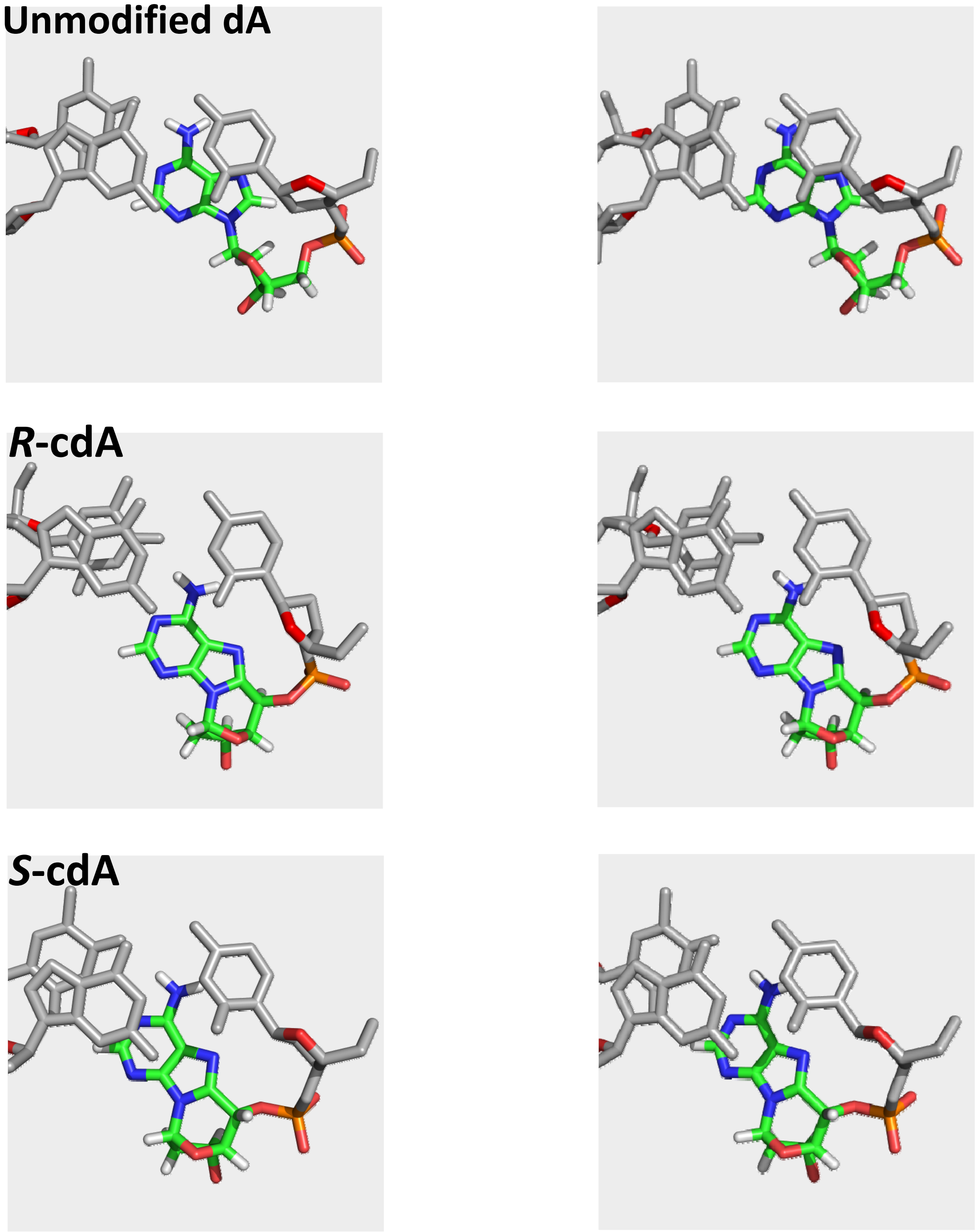

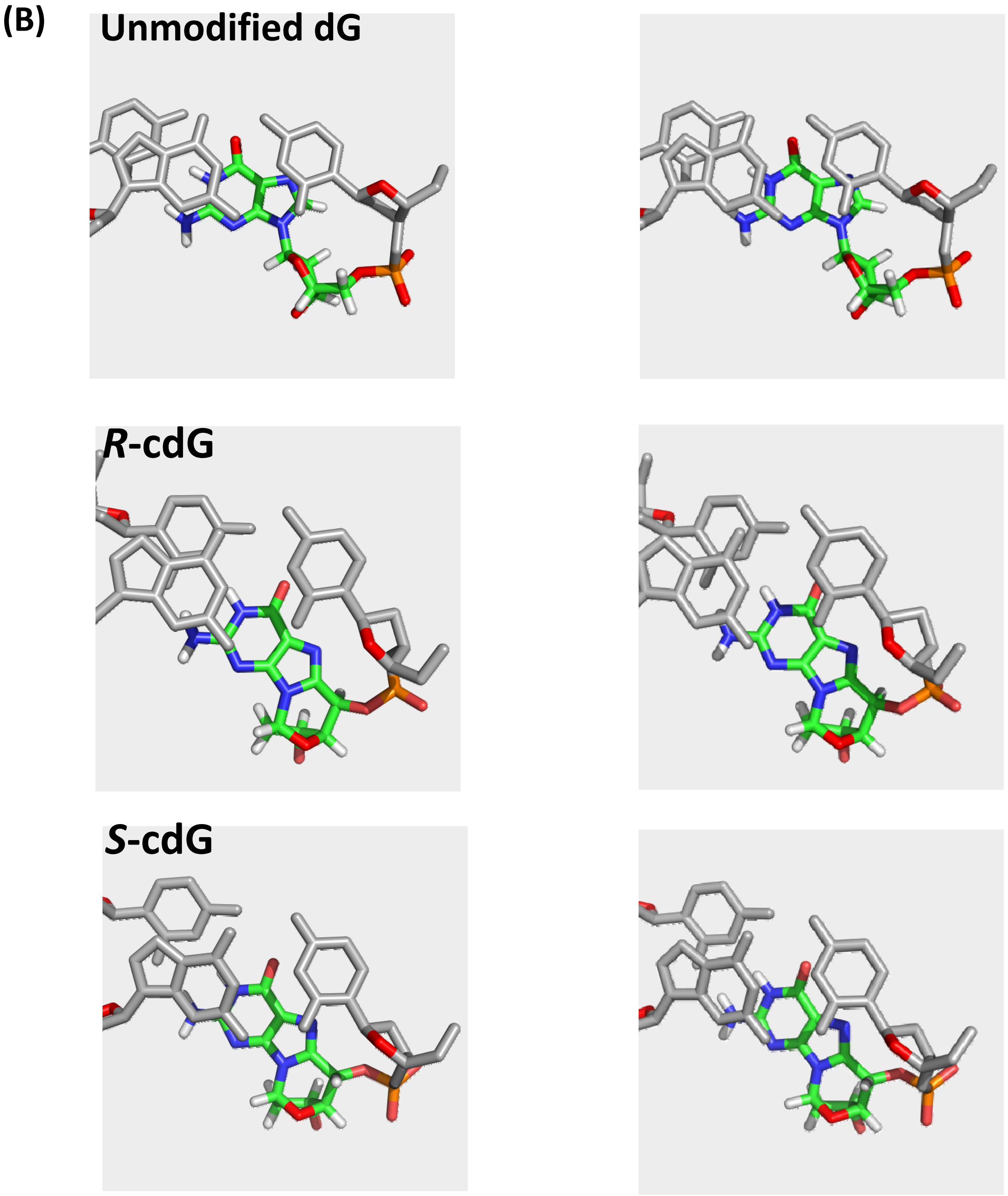

Figure S10. (A) Stereo views of the best representative structures looking down the helix axis of the (C5:G18)-(cdA:T17) base pair step shows the overtwisting, compared to its counterpart step in the unmodified case. (B) Stereo views of the best representative structures looking down the helix axis of the (C5:G18)-(cdG:C17) base pair step

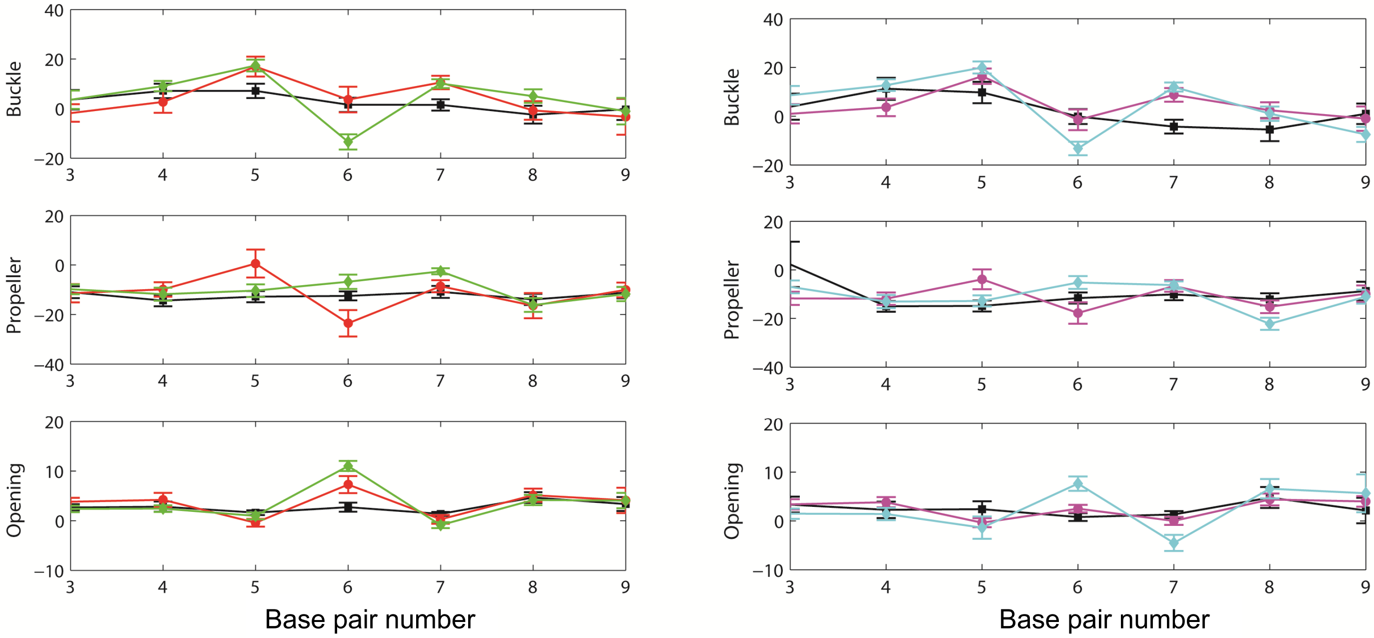

Buckle
Propeller
Opening
Base pair number
Buckle
Propeller
Opening
Base pair number

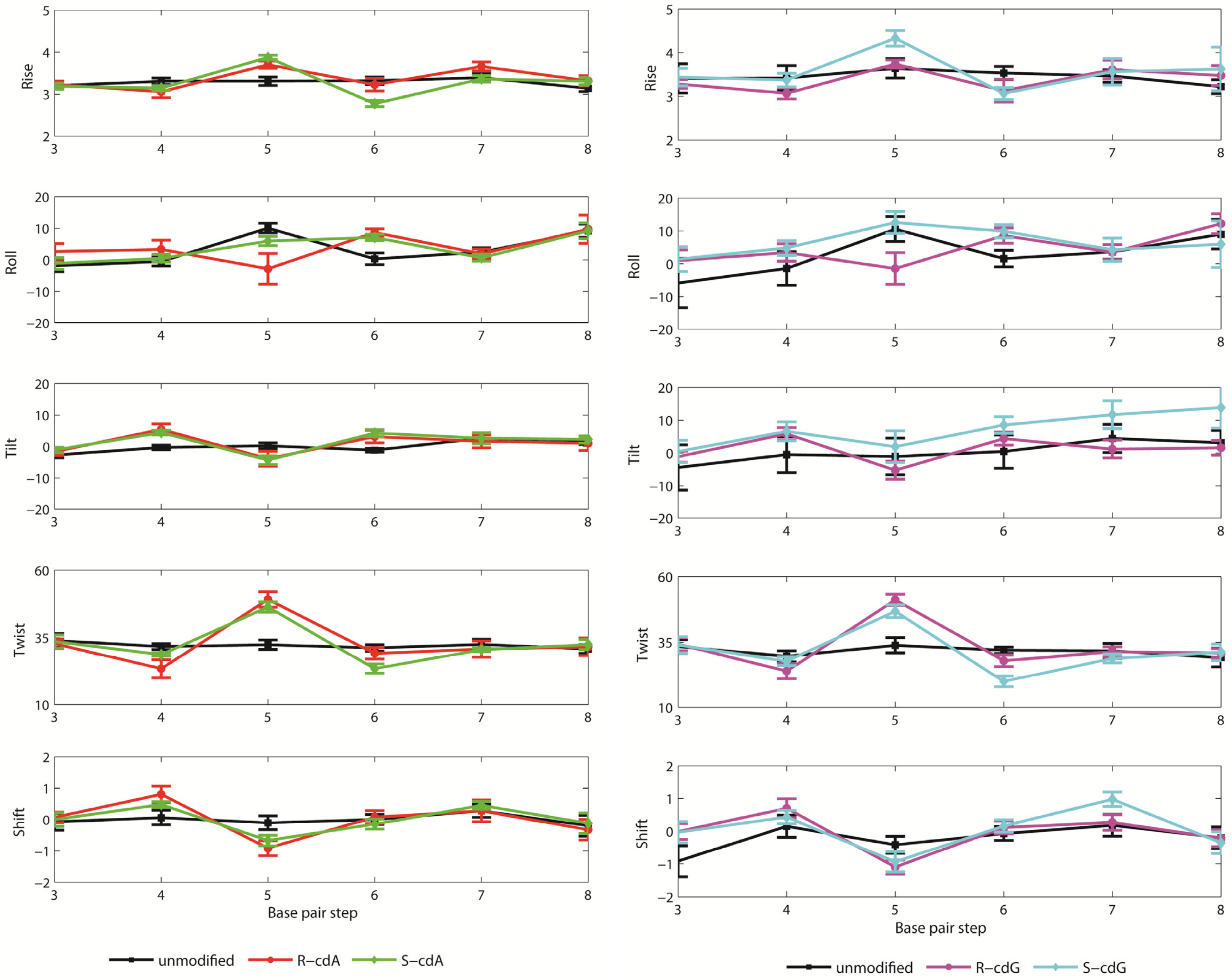

Figure S11. Ensemble average values of base pair parameters and standard deviations for the cyclo-modified duplexes and unmodified controls. For Buckle (degrees), Propeller (degrees), and Opening (degrees), A3:T20 is base pair number 3, A4:T19 is number 4, and so on; for Rise (Å), Roll (degrees), Tilt (degrees), Twist (degrees) and Shift (Å), A3:T20 to A4:T19 is step 3, A4:T19 to C5:G18 is step 2, and so on.

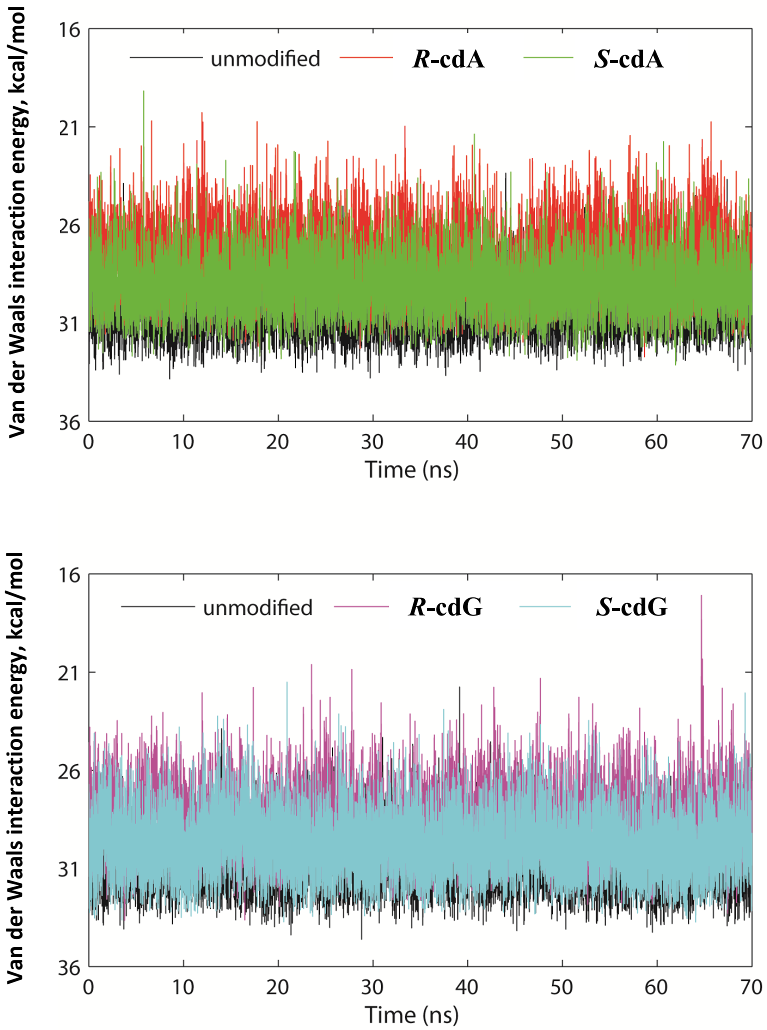

Figure S12. Time-dependent van der Waals interaction energies (kcal/mol) of the central trimer for each MD simulation.

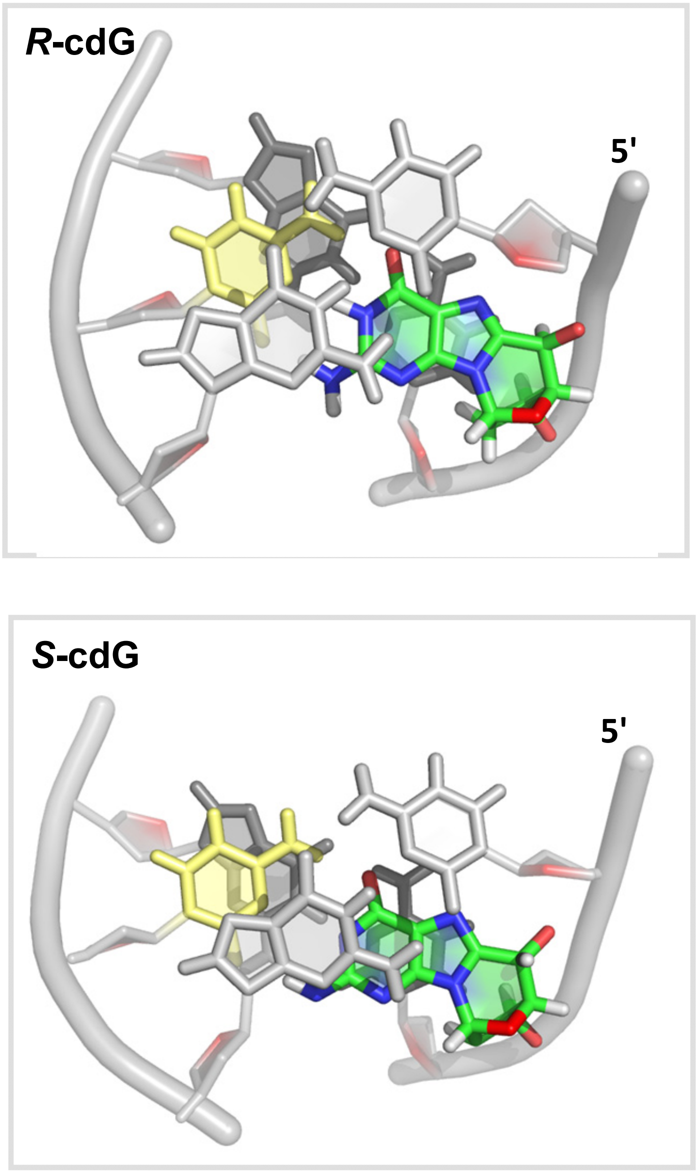

Figure S13. Best representative structures looking down the helix axis of the (C5:G18)-(cdG:C17)-(C7:G16) central trimer section show that the greater backbone distortions in the 5'*R*-stereoisomer lead to diminished stacking on the damaged strand for the 5'*R*-cdG, compared to the 5'*S*-cdG. Color code is the same as in Figure 8.

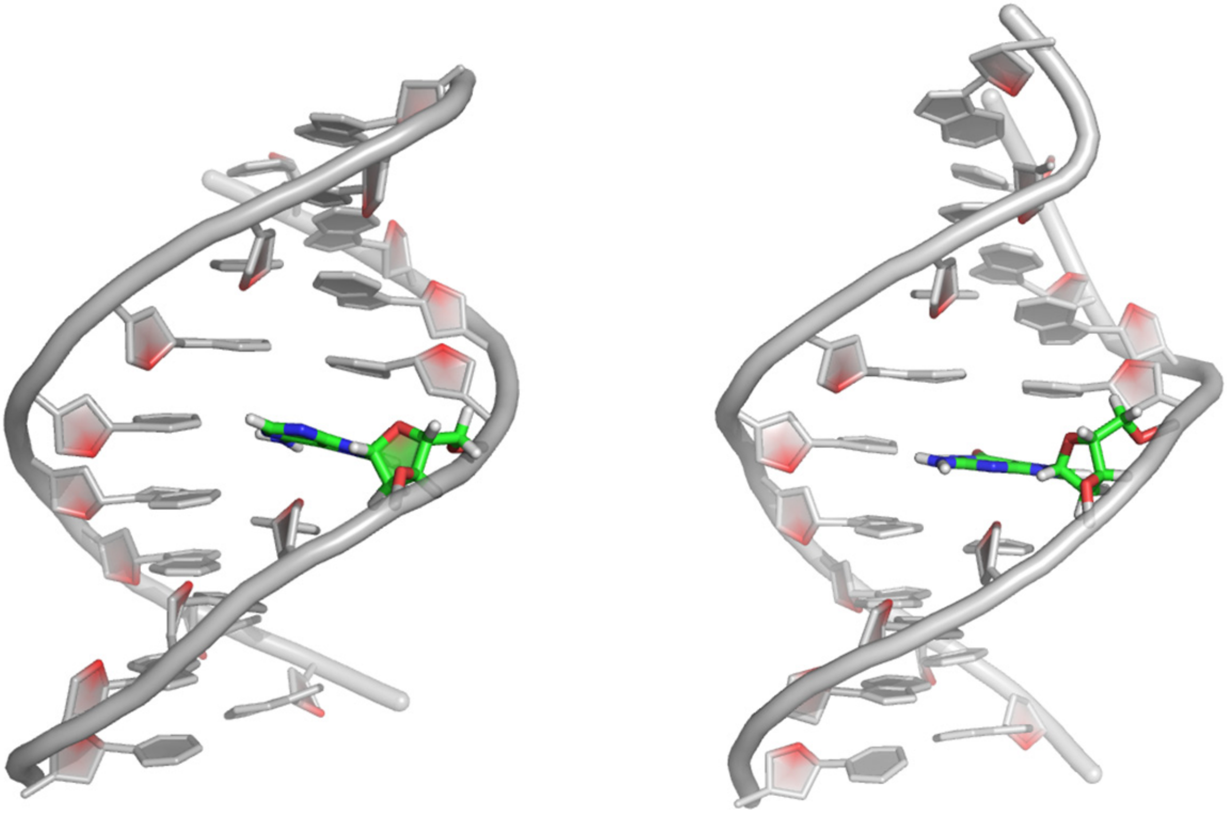

Unmodified dA **Unmodified dG**

Figure S14. Central 9-mers of the best representative structures from the MD simulations of the unmodified duplexes.

References

61. Case, D.A., Darden, T.A., Cheatham III, T.E., Simmerling, C.L., Wang, J., Duke, R.E., Luo, R., Walker, R.C., Zhang, W., Merz, K.M. *et al.* (2012). AMBER 12, University of California, San Francisco.
68. Flyvbjerg, H. and Petersen, H.G. (1989) Error-Estimates on Averages of Correlated Data. *J. Chem. Phys.*, **91**, 461-466.
69. Yang, W., Bitetti-Putzer, R. and Karplus, M. (2004) Free energy simulations: Use of reverse cumulative averaging to determine the equilibrated region and the time required for convergence. *J. Chem. Phys.*, **120**, 2618-2628.
89. Jorgensen, W.L., Chandrasekhar, J., Madura, J.D., Impey, R.W. and Klein, M.L. (1983) Comparison of simple potential functions for simulating liquid water. *J. Chem. Phys.*, **79**, 926-935.
90. Essmann, U., Perera, L., Berkowitz, M.L., Darden, T., Lee, H. and Pedersen, L.G. (1995) A Smooth Particle Mesh Ewald Method. *J. Chem. Phys.*, **103**, 8577-8593.
91. Darden, T., York, D. and Pedersen, L. (1993) Particle mesh Ewald - an N.LOG(N) method for Ewald sums in large systems. *J. Chem. Phys.*, **98**, 10089-10092.
92. Ryckaert, J.P., Ciccotti, G. and Berendsen, H.J.C. (1977) Numerical-Integration of Cartesian Equations of Motion of a System with Constraints - Molecular-Dynamics of N-Alkanes. *J. Comput. Phys.*, **23**, 327-341.
